# Untargeted and Targeted Lipidomics Unveil Dynamic Lipid Metabolism Alterations in Type 2 Diabetes

**DOI:** 10.3390/metabo14110610

**Published:** 2024-11-10

**Authors:** Li Feng, Bingshu He, Jianzhen Xia, Zhonghua Wang

**Affiliations:** 1 School of Agroforestry and Medicine, The Open University of China, Beijing 100039, China; fengli@ouchn.edu.cn; 2 Center for Imaging and Systems Biology, College of Life and Environmental Sciences, Minzu University of China, Beijing 100081, China; s161085@muc.edu.cn; 3 School Hospital, Minzu University of China, Beijing 100081, China; 2014007@muc.edu.cn

**Keywords:** type 2 diabetes, lipid metabolism, lipidomics, early diagnosis

## Abstract

Background: Type 2 diabetes mellitus (T2DM) is a complex metabolic disorder with a growing body of evidence suggesting the central role of lipid metabolism in its pathogenesis. However, the dynamic changes in lipid metabolism across different stages of T2DM remain understudied. Objective: This study aimed to elucidate the temporal alterations in lipid metabolism in T2DM using an integrated lipidomics approach. Method: Serum samples from 155 subjects were analyzed using LC-MS-based lipidomics, including untargeted and targeted approaches. Results: We identified significant alterations in 44 lipid metabolites in newly diagnosed T2DM patients and 29 in high-risk individuals, compared with healthy controls. Key metabolic pathways such as sphingomyelin, phosphatidylcholine, and sterol ester metabolism were disrupted, highlighting the involvement of insulin resistance and oxidative stress in T2DM progression. Moreover, 13 lipid metabolites exhibited diagnostic potential for T2DN, showing consistent trends of increase or decrease as the disease progressed. Conclusion: Our findings underscore the importance of lipid metabolism in T2D development and identify potential lipid biomarkers for early diagnosis and monitoring of disease progression, which contribute to paving the way for novel therapeutic strategies.

## 1. Introduction

Type 2 diabetes (T2D) is a complex metabolic disorder characterized by hyperglycemia resulting from impaired insulin secretion and/or insulin resistance. The pathogenesis of T2D is influenced by a complex interplay of genetic and environmental factors, and the exact molecular mechanisms remain to be fully elucidated [1]. One of the significant challenges associated with T2D is its often asymptomatic nature during the early stages, leading to delayed diagnosis and increased risk of complications. Research has consistently demonstrated the importance of early detection and intervention in mitigating the adverse health outcomes of T2D [2].

Lipid metabolism plays a pivotal role in T2D, with accumulating evidence suggesting its involvement in both the development and progression of the disease [3]. Lipids serve as essential components of cell membranes, energy storage molecules, and signaling mediators. Dysregulation of lipid metabolism, including alterations in lipid composition and signaling pathways, has been linked to insulin resistance and other metabolic abnormalities associated with T2D [4,5].

While previous studies have highlighted the role of lipid metabolism in T2D, a comprehensive understanding of the dynamic changes in lipid profiles throughout the disease process remains elusive. This knowledge is crucial for the development of sensitive biomarkers for early diagnosis, effective therapeutic strategies, and improved disease management. 

**Figure 1:**
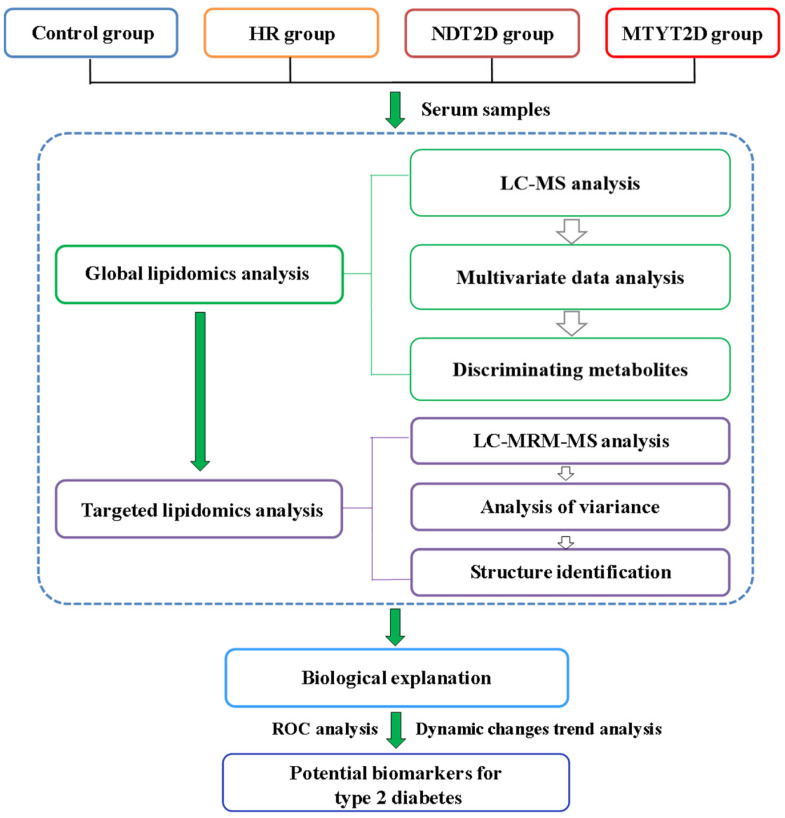
Research strategy for lipidomics analysis of type 2 diabetes. Control: healthy individuals; HR: high-risk individuals for type 2 diabetes; NDT2D: newly diagnosed type 2 diabetes patients; MTYT2D: type 2 diabetes patients diagnosed for more than two years.

In this study, we conducted a comprehensive lipidomics analysis of serum samples from individuals with T2D at different stages of disease progression to identify Dynamic alterations associated with T2D development. An untargeted lipidomic approach was employed to discover a broad range of lipid metabolites affected by the physiological and pathological states of the body. Subsequently, a targeted lipidomic approach was developed to further screen and validate the differential metabolites identified in the untargeted analysis, aiming to identify more reliable potential biomarkers. Multivariate statistical analysis, dynamic change trend analysis, and ROC analysis were employed to analyze the obtained data and uncover potential biomarkers for early diagnosis and disease monitoring. The research strategy is shown in Figure 1.

## 2. Materials and Methods

### 2.1. Chemicals and Reagents

HPLC-grade acetonitrile, isopropanol, methanol, and formic acid were obtained from Merck (Darmstadt, Germany). HPLC-grade ammonium formate and methyl tert-butyl ether, 17 lipid standards (including 2 fatty acids, 5 glycerolipids, 2 sphingolipids, 4 glycerophospholipids, 2 sterol lipids, and 2 prenol lipids) (Supplementary Materials Table S1), and internal standards LysoPC (17:0), PC (17:0/17:0), and TG (17:0/17:0/17:0), used in targeted analyses, were purchased from Sigma-Aldrich (St. Louis, MO, USA). Purified water was sourced from Wahaha (Hangzhou, China).

### 2.2. Study Subjects

A total of 155 male subjects aged 35–65 years were recruited and categorized into four groups: healthy controls (Control), high risk (HR), newly diagnosed type 2 diabetes (NDT2D), and more-than-two-year type 2 diabetes (MTYT2D). To minimize potential confounding factors related to hormonal fluctuations, only male subjects were included in this study. The inclusion of the MTYT2D group aimed to investigate how lipid metabolism evolves over time in individuals with a longer duration of T2DM. By studying patients with a chronic disease state, we can gain insights into potential biomarkers for monitoring disease progression and informing treatment strategies. The control group consisted of 40 healthy volunteers with normal physical examinations, laboratory test results, and no prior history of diabetes. The HR group included 40 individuals with a BMI ≥ 25, impaired glucose tolerance, or glycated hemoglobin values between 5.7% and 6.5% as determined by OGTT testing. The NDT2D group comprised 39 individuals recently diagnosed with type 2 diabetes who had not received any drug treatment. The MTYT2D group included 36 patients with a clinical diagnosis of type 2 diabetes for more than two years who had not undergone lipid-lowering treatment within three months prior to sampling. The general characteristics of the participants are summarized in Table 1. Fasting blood samples were collected from all subjects. Serum samples were immediately prepared by centrifuging the blood at 4000 rpm and stored at −80 °C until analysis. This study was approved by the Biological and Medical Ethics Committee of Minzu University of China (Approval No.: 2017-01).

### 2.3. Lipidomics Analysis

#### 2.3.1. Sample Preparation

Frozen serum samples were thawed at room temperature, vortexed for 30 s, and mixed. Thirty microliters (µL) of serum and 200 µL of methanol containing 1 µg/mL of internal standards (LysoPC (17:0), PC (17:0/17:0), and TG (17:0/17:0/17:0)) were added to a 1.5 mL Eppendorf tube and vortexed for 20 s. Subsequently, 660 µL of methyl tert-butyl ether and 150 µL of water were added, followed by vortexing for 5 min. After standing for 5 min, the samples were centrifuged at 8 °C and 10,000 rpm for 5 min. Six hundred microliters of the upper organic phase were concentrated to dryness in a vacuum centrifuge concentrator (SPD121P, Thermo Scientific, Waltham, MA, USA) at 50 °C. The evaporated material was reconstituted with 600 µL of an acetonitrile/isopropanol/water (65:30:5, *v*/*v*/*v*) mixture. After centrifuging at 8 °C and 15,000 rpm for 10 min, 10 µL of the supernatant was injected into the UPLC-MS/MS system for analysis. To ensure experimental quality and reproducibility, 10 µL of a pooled serum sample (QC sample) was processed alongside the actual samples.

#### 2.3.2. Untargeted Lipidomics Analysis

The untargeted lipidomics analysis of serum samples was performed using a quadrupole electrostatic field orbital trap high-resolution mass spectrometry system (Q Exactive, Thermo Scientific Technologies, Waltham, MA, USA) equipped with an ESI source and Xcalibur (version 2.2, Thermo Scientific Technologies) data processing system. Data were acquired in both positive and negative ion modes under the following instrumental conditions: ESI spray voltage 3.5/−3.5 kV; capillary temperature, 450 °C; sheath gas flow rate, 60 arbitrary units (arb); aux gas flow rate, 30 arb; sweep gas flow rate, 0 arb; capillary temperature, 380 °C; aux gas heater temperature, 300 °C; and scan range, *m*/*z* 10–1200. Nitrogen gas was used for nebulizing and drying. To obtain the MS/MS spectra of metabolites, a data-dependent secondary scanning mode (ddMS2) was employed with a collision energy (CE) of 20, 35, 50 eV or −20, −35, −50 eV, respectively. Data acquisition and processing were performed using Xcalibur(version 2.2).

Chromatographic separation was performed on an Ascentis Express C18 (1.7 µm, 10 cm × 2.1 mm; Sigma-Aldrich, USA) column using a Waters ACQUITY UPLC system (Waters Corporation, Milford, MA, USA). The column temperature was maintained at 45 °C, and a mobile phase consisting of solvent A (water containing 0.1% formic acid and 10 mM ammonium formate) and solvent B (isopropanol/acetonitrile, 9:1, *v*/*v*, containing 0.1% formic acid and 10 mM ammonium formate) was used at a flow rate of 260 µL/min. The gradient conditions were as follows: 0–1.5 min, 32% solvent B; 1.5–4 min, linear gradient to 45% solvent B; 4–5 min, linear gradient to 52% solvent B; 5–8 min, linear gradient to 58% solvent B; 8–11 min, linear gradient to 66% solvent B; 11–14 min, linear gradient to 70% solvent B; 14–18 min, linear gradient to 75% solvent B; 18–21 min, linear gradient to 97% solvent B; 21–25 min, 97% solvent B; 25.1–25.1 min, linear gradient to 32% solvent B; and 25.1–30 min, 32% solvent B.

#### 2.3.3. Targeted Lipidomics Analysis

Targeted lipidomics was performed using a quadrupole linear ion trap tandem mass spectrometer (QTRAPTM 5500, Applied Biosystems/MDS SCIEX, Foster City, CA, USA) equipped with an ESI source. ESI detection was performed in both positive and negative ion modes using a multiple reaction monitoring (MRM) scanning mode. Nitrogen gas was used in various gas pathways with the following specific parameters: Ionspray Voltage, 5/−4.5 kV; Curtain Gas, 45/40 psi; Collision Gas, Medium; Temperature, 500 °C; Nebulizer Gas, 60 arbitrary units (arb); Source Gas 2, 50 arb; Declustering Potential, 50/−50 V; and Focusing Potential, 15/−15 V. Data acquisition and processing were performed using Analyst software (version 1.5.1, Applied Biosystems/MDS SCIEX). Chromatographic separation was the same as that used for the untargeted lipidomics analysis.

#### 2.3.4. Raw Data Processing

For the untargeted lipidomics analysis, UPLC-MS raw data files were converted to the mzXML format using the ProteoWizard msconvert tool (http://proteowizard.sourceforge.net/, accessed on 10 Novermber 2012). Peak finding, filtering, alignment, and scaling were subsequently performed using open-source XCMS software (version 4.3.3) [6] and CAMERA (version 1.20.0) [7] operated within the R statistical software (version 3.6.2) [8]. The parameters for the detailed data preprocessing are available in the Supplementary Materials. 

For the targeted lipidomics analysis, raw data files were processed using Analyst software (version 1.5.1). Peak detection, integration, and quantification were performed using the software’s built-in algorithms. The peak areas of targeted lipid metabolites were normalized to internal standards to account for variations in sample preparation and instrument response.

### 2.4. Statistical Analysis

Total ion current (TIC) normalization was applied to the LC-MS data prior to statistical analysis. The normality of the numerical data was assessed using the one-sample Kolmogorov–Smirnov test. A multivariate analysis of the untargeted lipidomic data obtained from the UPLC-MS analysis, including principal component analysis (PCA) and orthogonal partial least-squares discriminant analysis (OPLS-DA), was performed using SIMCA-P software (Umetrics AB, Umea, Sweden, version 14.0). Independent two-sample *t*-tests were used to compare the differences in numerical variables. All statistical analyses were conducted in the R statistical system, except for the multivariate analyses.

## 3. Results

### 3.1. Untargeted Lipidomics Analysis 

#### 3.1.1. UPLC-MS Analysis 

**Figure 2:**
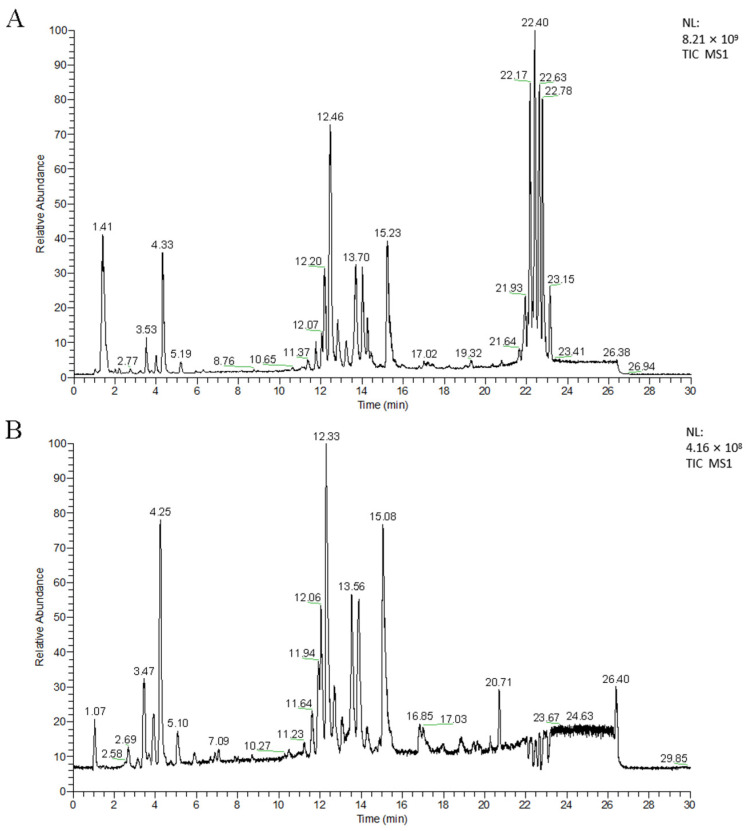
Typical TICs obtained from serum sample via (**A**) LC-(+)ESIMS and (**B**) LC-(−)ESIMS analyses.

Representative total ion chromatograms (TIC) acquired from the serum samples of the different groups using UPLC-MS analysis in positive ion mode (UPLC-(+)ESI-MS) and negative ion mode (UPLC-(−)ESI-MS) are shown in Figure 2. From Figure 2, it can be seen that different types of lipids in the serum could be separated well in the elution time of 30 min due to the minor particles (1.7 µm) of the column packing and excellent separation ability of the UPLC system. In order to evaluate the stability of the chromatographic system, retention time variation plots were plotted for LC-(±) ESIMS data of all 155 test samples by using R language package XCMS. Positive retention time deviations indicated that the retention time of metabolites in the sample was greater than the standard retention time, while negative retention time deviations indicated that the retention time of metabolites in the sample was less than the standard retention time. The results showed that the retention time variation in the vast majority of the LC-(±) ESIMS spectral data was within ±15 s, indicating that the chromatographic system was relatively stable and reliable in the large-scale analysis of the serum samples (Supplementary Materials Figure S1). In addition, in order to monitor the stability of the analysis system and ensure the reliability of data quality, the QC samples and the test samples were preprocessed simultaneously in the experiment. At the beginning of each analysis batch, three consecutive QC sample measurements were conducted to stabilize the analysis system. Subsequently, after every 10 samples to be tested in the detection sequence, one QC sample was tested, and then a PCA was performed on all QC samples. Projection results of the LC-(+) ESIMS data matrix of all QC samples on the first principal component (Supplementary Materials Figure S2A) and second principal component (Supplementary Materials Figure S2B) after the PCA showed that the relative deviation of peak areas for all QC samples was within 2SD. Similar results were obtained in the LC-(−) ESIMS data matrix (Supplementary Materials Figure S3A,B), which indicated good stability of the analysis system during large-scale analysis. All the above mentioned results indicate that the significant differences observed between the groups were primarily due to genuine metabolite changes rather than analytical artifacts. By employing an optimized spectral data analysis protocol, 4613 and 1995 features were extracted from the UPLC-MS analysis via ESI in positive and negative ion modes (UHPLC-(±)ESI-MS), respectively.

#### 3.1.2. OPLS-DA Analysis 

**Figure 3:**
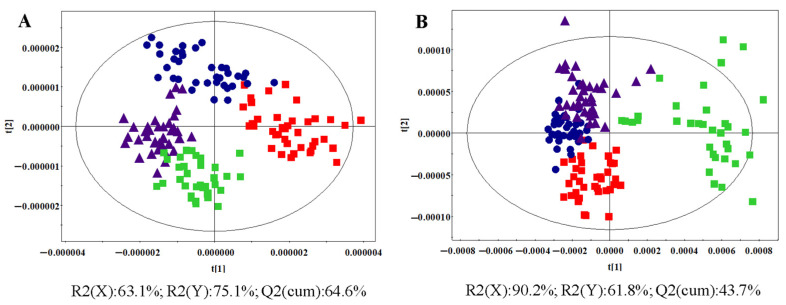
OPLS-DA score plots based on the LC-(+)ESI-MS data (**A**) (R2X: 63.1%; R2(Y): 75.1%; Q2(cum): 64.6%) and the LC-(−) ESI-MS data (**B**) (R2X: 90.2%; R2(Y): 61.8%; Q2(cum): 43.7%) from all the groups (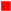: control group, 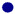: HR group, 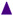: NDT2D group, 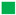: MTYT2D group).

To investigate global lipidome differences between the groups, multivariate statistical analyses of the UPLC-MS data were performed. The OPLS-DA scatter plot (Figure 3A) demonstrated clear separation of the different groups based on the 4613 peaks detected by UPLC-(+)ESI-MS. The quality of the OPLS-DA model was assessed using the R2(Y) and Q2(cum) parameters, which represented the fitness and prediction capability, respectively. With one predictive and two orthogonal components, the OPLS-DA model exhibited an R2(X) value of 63.1%, an R2(Y) value of 75.1%, and a Q2(cum) value of 64.6%. Similarly, the OPLS-DA model for the negative data demonstrated an R2(X) value of 90.2%, an R2(Y) value of 61.8%, and a Q2(cum) value of 43.7% across one predictive and two orthogonal components (Figure 3B).

**Figure 4:**
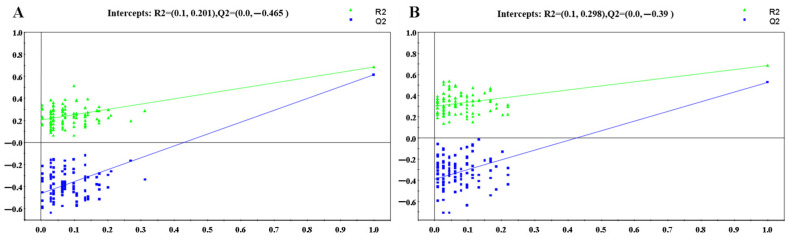
PLS-DA validation plots of 100 random permutations based on the LC-(+)ESIMS data (**A**) (R2 = (0.0, 0.201), Q2 = (0.0, −0.465)) and LC-(−)ESIMS data (**B**) (R2 = (0.0, 0.298), Q2 = (0.0, −0.39)) from all the groups.

Furthermore, statistical validation of the corresponding PLS-DA model was conducted to evaluate overfitting of the multivariate statistical model. Permutation testing with 100 permutations generated intercepts of R2 = 0.201 and Q2 = −0.465 for positive mode (Figure 4A) and R2 = 0.298 and Q2 = −0.39 for negative mode (Figure 4B), respectively. These results indicate that the OPLS-DA models derived from the UPLC-(±)ESI-MS data were statistically valid and acceptable.

**Figure 5:**
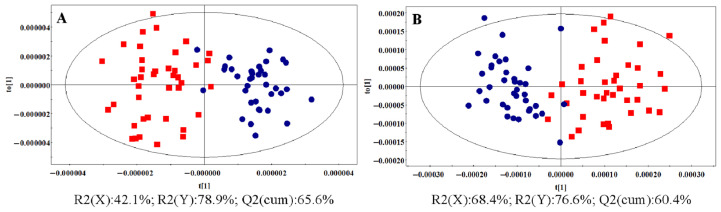
OPLS-DA score plots based on the LC-(+)ESIMS data (**A**) (R2X: 42.1%; R2(Y): 78.9%; Q2(cum): 65.6%) and the LC-(−) ESIMS data (**B**) (R2X: 68.4%; R2(Y): 76.6%; Q2(cum): 60.4%) from the control and HR groups (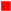: control group, 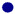: HR group).

OPLS-DA was also applied to the UPLC-(±)ESI-MS data of the control and HR groups, demonstrating a separation trend. Using three latent variables, the R2(X), R2(Y), and Q2(cum) values were 42.1, 78.9, and 65.6%, respectively, for the UPLC-(+)ESI-MS data (Figure 5A) and 68.4, 76.6, and 60.4%, respectively, for the UPLC-(−)ESI-MS data (Figure 5B).

**Figure 6:**
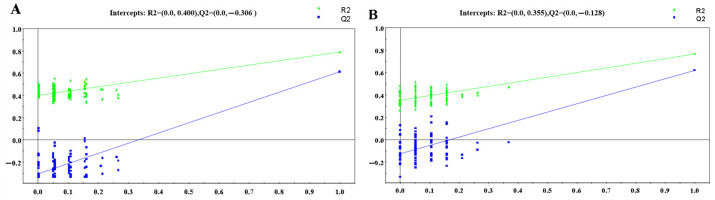
PLS-DA validation plots of 100 random permutations based on the LC-(+)ESIMS data (**A**) (R2 = (0.0, 0.400), Q2 = (0.0, −0.306)) and the LC-(−)ESIMS data (**B**) (R2 = (0.0, 0.355), Q2 = (0.0, −0.128)) from the control and HR groups.

The Q2 intercepts for the corresponding PLS-DA models were less than 0.4, indicating that the OPLS-DA models were not overfitted (Figure 6). Similarly, OPLS-DA analysis of the UPLC-(±)ESI-MS data from the control and NDT2D groups, as well as the control and MTYT2D groups, revealed good separation and were statistically valid and acceptable (Supplementary Materials Figures S4–S7).

#### 3.1.3. Screening of Differential Metabolites

To identify discriminating metabolites among the thousands of variables, the variable importance in projection (VIP) value of each peak was calculated to assess the contributions of the X variables to the OPLS-DA model. Using a VIP threshold of >1.0 and a *p*-value < 0.05 in Student’s *t*-tests, 118 and 115 differential metabolites were screened in positive ion detection mode for the control group versus the HR group and the control group versus the NDT2D group, respectively (Supplementary Materials Tables S2 and S3). In negative ion detection mode, 63 and 70 differential metabolites were identified for the control group versus the HR group and the control group versus the NDT2D group, respectively (Supplementary Materials Tables S4 and S5).

### 3.2. Targeted Validation of Differential Metabolites

**Figure 7:**
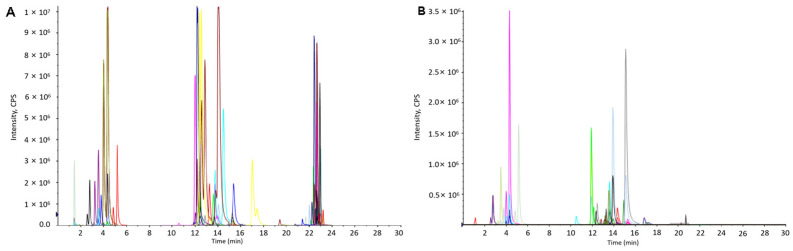
The typical extract ions chromatograms (XIC) of the LC-MRM-MS analysis from QC sample: (**A**) positive ion mode and (**B**) negative ion mode (each colored line is a extracted ion chromatographic peak).

Targeted LC-MS/MS using multiple reaction monitoring (MRM) was performed to validate the differential metabolites identified in the untargeted lipidomics analysis. Optimized parameters, including parent and product ions, declustering potential (DP), and collision energy (CE), were used to establish 115 MRM ion pairs in positive mode and 56 in negative mode (see Supplementary Materials Tables S6 and S7). Internal standards—LysoPC (17:0), PC (17:0/17:0), and TG (17:0/17:0/17:0)—were included to ensure accuracy, with optimized DP and CE values. Sample preparation and chromatographic conditions were consistent with the untargeted analysis. Typical extracted ion chromatograms (XICs) from the QC sample are shown in Figure 7.

The LC-MRM-MS method was applied to the serum samples, and the data were analyzed using an independent sample *t*-test. In the comparison between the NDT2D and control groups, 49 of 185 differential metabolites showed no significant differences (*p* > 0.05), leaving 136. Similarly, 63 of 181 differential metabolites from the HR vs. control group comparison were excluded (*p* > 0.05), leaving 118 for further structural characterization in subsequent analyses.

### 3.3. Structural Identification of Differential Metabolites

Building on the prior research conducted in our laboratory, this study followed a systematic approach to identify the structures of differential metabolites. First, the quasi-molecular ions, adduct ions, fragment ions, isotope ions, and adduct ions of the differential metabolites, such as [M+H]^+^, [M−H]^−^, or [M+Na]^+^, were analyzed and excluded manually using our lab’s homemade metabolites database and referencing annotation information of R language package CAMERA. Then, using the precise mass of the quasi-molecular ions and applying the “7 Golden Rules” [9], the possible molecular compositions were inferred. Next, high-resolution MS and MS/MS spectra, along with the fragmentation patterns of various metabolites, were analyzed in conjunction with online databases such as LipidMaps, HMDB, and literature searches. Finally, for metabolites with available standard samples, their structures were confirmed by comparing chromatographic retention times and MS/MS fragmentation patterns with those of the standards.

Following the above approach, 29 differential metabolites were identified to distinguish the control group from the HR group, including 7 sphingosine metabolites, 2 sphingomyelin metabolites, 3 lysophosphatidylcholine, 11 phosphatidylcholine, 5 triacylglycerol, and 1 cholesterol ester (Table 2). In addition, 44 differential metabolites were identified to distinguish the control group from the NDT2D group, including 7 sphingosine metabolites, 3 sphingomyelin metabolites, 1 cerebral phospholipid metabolite, 3 lysophosphatidylcholine, 10 phosphatidylcholine, 3 phosphatidylethanolamine, 1 diacylglycerol, 12 triacylglycerol, and 4 cholesterol esters (Table 3). 

Notably, among the 29 differential metabolites distinguishing the control group from the HR group, 20 also differentiated the control group from the NDT2D group. The structures of nine metabolites, including LysoPC (16:0), LysoPC (18:0), LysoPC (18:1), PC (16:0/18:2), PC (18:0/20:4), PC (18:0/18:2), PC (16:0/18:1), PC (18:0/22:6), and SM (d18:1/16:0), were confirmed through comparative analysis with standard samples. However, due to the unavailability of standard samples for other compounds, the structures of the remaining 44 differential metabolites have not been fully confirmed.

## 4. Discussion

### 4.1. Dynamic Trend Analysis of Differential Metabolites

In this study, we collected clinical serum samples from patients with type 2 diabetes at different stages to carefully investigate the dynamic change trend of lipid metabolites in the development of type 2 diabetes. The potential biological significance was analyzed by combining the dynamic changes in differential metabolites among different experimental groups.

#### 4.1.1. Dynamic Change in Sphingolipids Metabolism

**Figure 8:**
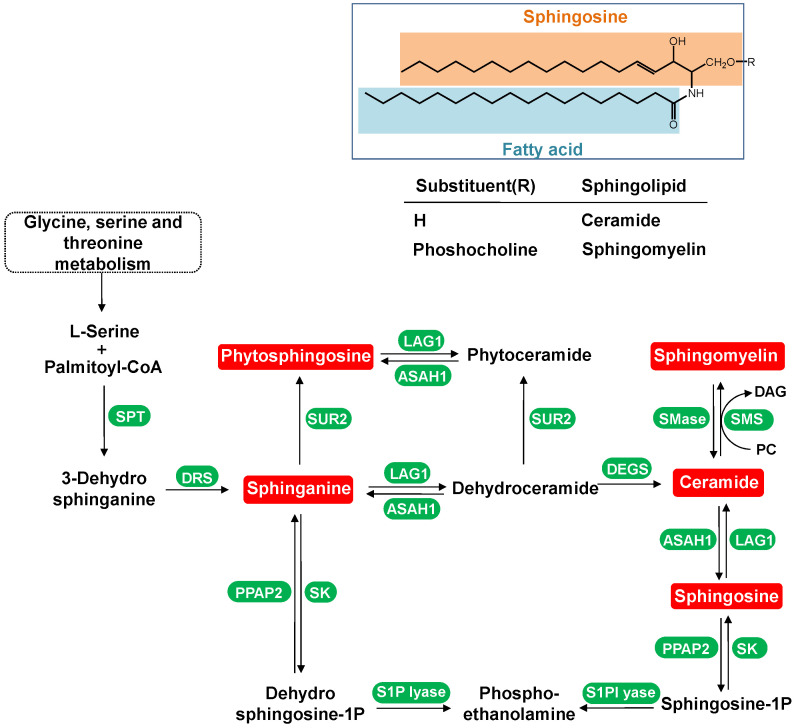
Scheme of sphingolipid biosynthesis and metabolism. ASAH1, acid ceramidase; DEGS, sphingolipid delta-4 desaturase; DRS, 3-dihydrosphingosine reductase; LAG1, Acyl-CoA-dependent ceramide synthase; PPAP2, phosphatidate phosphatase; S1Plyase, sphinganine-1-phosphate aldolase; SK, sphingosine kinase; SPT, serine palmitoyl transferase; SUR2, sphinganine C4-monooxygenase; SMS, sphingomyelin synthase; SMase, sphingomyelin phosphodiesterase.The items highlighted in red are important sphingolipid metabolites and the items highlighted in green are metabolic enzymes associated with these important sphingolipid metabolites.

Sphingolipid metabolites are crucial components of cell membranes and play roles in processes such as cell growth, differentiation, aging, and apoptosis [10]. Their synthesis involves the conversion of serine and palmitoyl-CoA into sphingosine, which then forms ceramide (Cer). Ceramide can be further phosphorylated to produce sphingomyelin (SM) or glycosphingolipids or reconverted into sphingosine by hydrolytic enzymes. Dihydrosphingosine and sphingosine are key units in complex sphingolipid synthesis (Figure 8). Studies have shown decreased levels of dihydrosphingosine and sphingosine in the serum of type 2 diabetes (T2D) and diabetic nephropathy patients [11,12]. Ceramides, important signaling molecules in sphingolipid metabolism, reduce insulin sensitivity by inhibiting the insulin pathway and GLUT4 translocation [13]. 

Elevated ceramide levels have been linked to insulin resistance and TNF-α-induced insulin desensitization [14]. In pre-diabetic patients, increased neurophospholipase activity leads to reduced sphingomyelin and increased ceramide, negatively correlating with insulin sensitivity [15].

**Figure 9:**
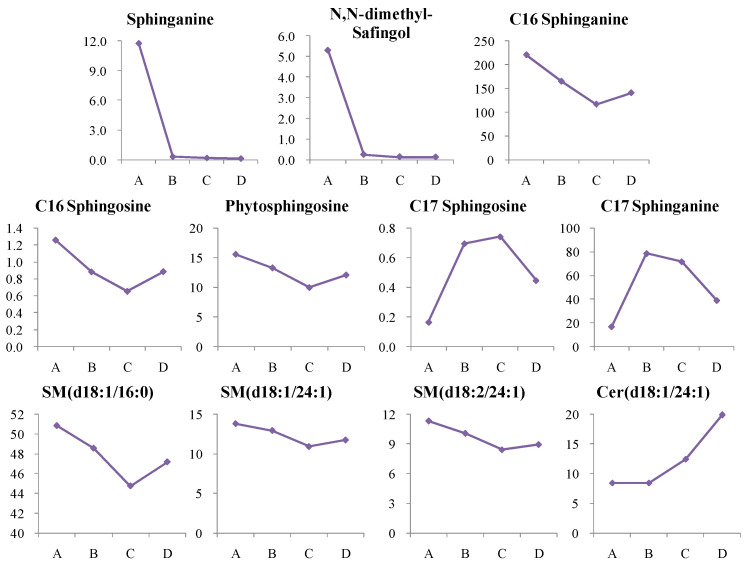
Change trends of sphingosines and their derivatives in different groups. The *x* axis is the experimental group. A, healthy control; B, high-risk population for type 2 diabetes; C, newly diagnosed type 2 diabetes; D, type 2 diabetes diagnosed for more than two years. The *y* axis is the relative concentration.

In this study, 11 sphingolipid metabolites were significantly altered in newly diagnosed T2D patients, with 8 reduced and 3 elevated. Except for Cer (d18:1/24:1), these metabolites showed similar trends in high-risk groups (*p* < 0.05). After more than two years of treatment, most metabolite levels normalized, except for Cer (d18:1/24:1) which continued to rise, indicating persistent insulin resistance despite treatment (Figure 9). The reduction in dihydrosphingosine and sphingosine levels may indicate impaired sphingolipid metabolism, which is crucial for insulin signaling and cellular function [11,12]. Elevated levels of ceramide, particularly Cer (d18:1/24:1), suggest a shift toward insulin resistance, as ceramides can inhibit insulin receptor activity and GLUT4 translocation, impairing glucose uptake [13]. 

While most sphingolipid levels normalized after two years of treatment, the persistent elevation of Cer (d18:1/24:1) highlights ongoing metabolic dysfunction and chronic inflammation. 

#### 4.1.2. Dynamic Change in Lysophosphatidylcholine (LysoPC) Metabolism

Lysophosphatidylcholine (LysoPC) is produced from phospholipids through the action of phospholipase A2 (LPA2) or lecithin cholesterol acyltransferase (LCAT) and plays a key role in regulating cell proliferation, tumor infiltration, and inflammation [16]. Its role in diabetes remains unclear. Some studies report elevated LysoPC levels in the plasma of obese individuals and type 2 diabetes (T2D) patients, linking it to insulin resistance and chronic inflammation [17]. However, other studies suggest that LysoPC levels are reduced in patients with impaired fasting glucose, T2D, and in animal models of obesity and insulin resistance, indicating that LysoPC may increase GLUT4 expression on adipocyte membranes, enhancing glucose uptake and improving glucose metabolism [18].

**Figure 10:**
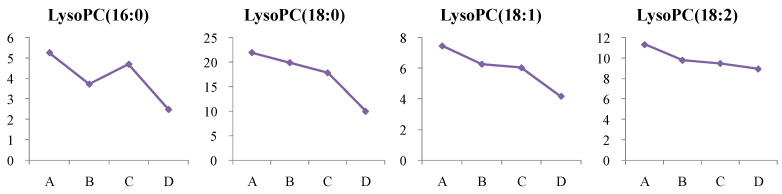
Change trends of Lyso PC (16:0), Lyso PC (18:0), Lyso PC (18:1), and Lyso PC (18:1) in different groups. The *x* axis is the experimental group. A, healthy control; B, high-risk population for type 2 diabetes; C, newly diagnosed type 2 diabetes; D, type 2 diabetes diagnosed for more than two years. The *y* axis is the relative concentration.

In this study, LysoPC (18:0), LysoPC (18:1), and LysoPC (18:2) were significantly reduced in newly diagnosed T2D patients, while LysoPC (16:0), LysoPC (18:0), and LysoPC (18:1) were reduced in the high-risk group (*p* < 0.05). After more than two years of treatment, these metabolites continued to decrease, suggesting that the treatment did not significantly restore LysoPC levels (Figure 10). 

The significant reduction in LysoPC levels in newly diagnosed type 2 diabetes (T2D) patients suggests disruptions in lipid signaling that may impair insulin sensitivity and glucose uptake [18]. The decreased levels of LysoPC (18:0), LysoPC (18:1), and LysoPC (18:2) could contribute to the insulin resistance observed in T2D, as LysoPC is known to enhance GLUT4 expression on adipocyte membranes [19].

The persistent decline in LysoPC levels after two years of treatment indicates ongoing dysregulation of lipid metabolism, potentially exacerbating inflammation and further hindering insulin signaling. This could lead to reduced glucose uptake and worsening glycemic control.

#### 4.1.3. Dynamic Change in Phosphatidylcholine (PC) Metabolism

Phosphatidylcholine (PC), also known as lecithin, is a key component of cell membranes and plays a role in cellular signal transduction [20]. It is the main lipid in human serum and lipoproteins, primarily synthesized in the liver, with dietary choline as a crucial ingredient. Diacyl phosphatidylcholine aids in the secretion of triglyceride-rich very low-density lipoprotein (VLDL) in the liver, while monoacyl phosphatidylcholine has antioxidant properties that protect serum lipoproteins from oxidation [21]. In choline-deficient mice, high-fat diets lead to liver fat accumulation due to reduced PC synthesis, though glucose tolerance improves [22]. Impaired PC synthesis also reduces plasma triglyceride and cholesterol ester levels [23].

**Figure 11:**
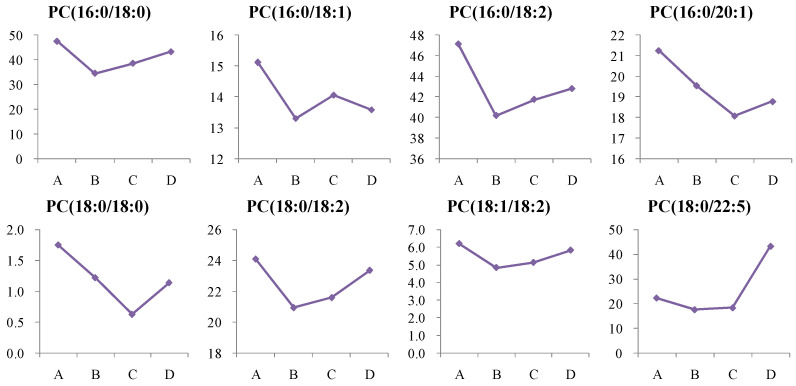
Change trends of phosphatidylcholine (PC) in different groups. The *x* axis is the experimental group. A, healthy control; B, high-risk population for type 2 diabetes; C, newly diagnosed type 2 diabetes; D, type 2 diabetes diagnosed for more than two years. The *y* axis is the relative concentration.

In this study, we observed a significant reduction in various PC levels in newly diagnosed T2D patients and high-risk individuals, indicating altered PC metabolism in both groups. Notably, after more than two years of treatment, PC levels in patients returned to normal, likely due to the effects of the medication (Figure 11).

The significant reduction in phosphatidylcholine (PC) levels in newly diagnosed type 2 diabetes (T2D) patients and high-risk individuals indicates disrupted lipid metabolism, which may affect membrane integrity and insulin signaling [20]. Lower PC levels could impair the secretion of triglyceride-rich very low-density lipoprotein (VLDL), contributing to dyslipidemia often seen in T2D and increasing cardiovascular risk [23].

The normalization of PC levels after more than two years of treatment suggests that therapeutic interventions can effectively restore PC homeostasis, potentially improving metabolic health. However, further investigation is needed to understand the mechanisms behind these changes and their implications for treatment strategies.

#### 4.1.4. Dynamic Change in Phosphatidylethanolamine (PE) Metabolism

Phosphatidylethanolamine (PE), a key component of cell membranes, is abundant in brain tissue and plays a role in membrane fusion, cell division, autophagy, and apoptosis [24]. It is mainly synthesized in mitochondria through enzymatic reactions using phosphatidylethanolamine and diacylglycerol. PE is the second most abundant lipid in human serum after phosphatidylcholine and is involved in the regulation of very low-density lipoprotein (VLDL) secretion in the liver [25]. Although PE levels in type 2 diabetes (T2D) patients have been reported to change significantly, the exact mechanisms remain unclear [26].

**Figure 12:**
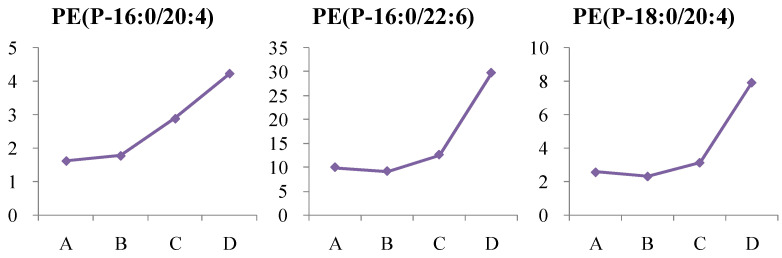
Change trends of phosphatidylethanolamine (PE) in different groups. The *x* axis is the experimental group. A, healthy control; B, high-risk population for type 2 diabetes; C, newly diagnosed type 2 diabetes; D, type 2 diabetes diagnosed for more than two years. The *y* axis is the relative concentration.

In this study, PE (P-16:0/20:4), PE (P-16:0/22:6), and PE (P-18:0/20:4) were significantly elevated in newly diagnosed T2D patients, while no significant changes were observed in the high-risk group. PE levels further increased in patients diagnosed for more than two years, suggesting that these metabolites are linked to disease progression, with medication showing limited effects on these indicators (Figure 12).

The significant elevation of phosphatidylethanolamine (PE) levels, particularly PE (P-16:0/20:4), PE (P-16:0/22:6), and PE (P-18:0/20:4), in newly diagnosed type 2 diabetes (T2D) patients suggests alterations in lipid metabolism that may indicate increased cellular activity related to membrane dynamics and autophagy [24].

The continued rise in PE levels after two years of diagnosis suggests a link to disease progression, potentially reflecting an adaptive response to metabolic stress or inflammation. The lack of significant changes in the high-risk group indicates that these alterations may be specific to T2D rather than risk factors.

The limited effect of medication on PE levels raises concerns about the ability of current treatments to normalize lipid metabolism. These findings highlight the relevance of PE in T2D pathophysiology and suggest that further investigation into its role could provide valuable insights into metabolic health and disease progression.

#### 4.1.5. Dynamic Change in Triglycerides (TG) Metabolism

Triglyceride deposition is a key factor in insulin resistance and pancreatic beta cell damage [27]. Elevated fasting triglyceride levels are an independent risk factor for type 2 diabetes (T2D) [28]. The relationship between triglycerides and T2D depends on fatty acid composition: short-chain, saturated fatty acid triglycerides are positively associated with T2D, while long-chain, unsaturated fatty acid triglycerides are negatively associated [29]. Weight loss through dietary control can reduce short-chain triglycerides and improve insulin sensitivity in obese individuals [30]. However, triglyceride levels are easily influenced by diet and lifestyle, limiting their predictive value for T2D risk [31].

**Figure 13:**
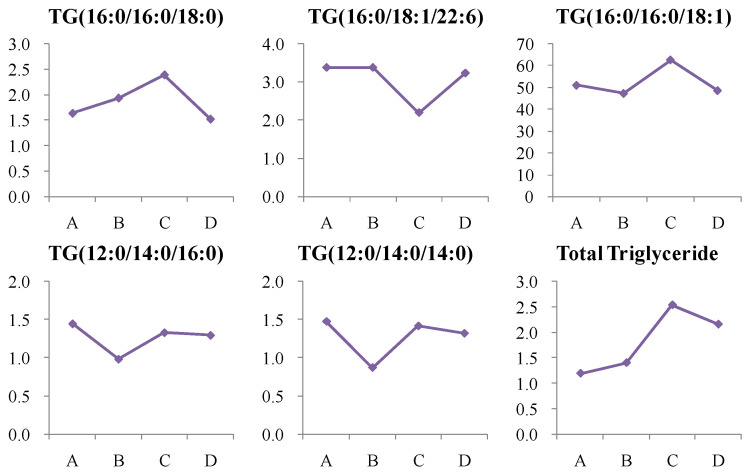
Change trends of triglyceride (TG) in different groups. The *x* axis is the experimental group. A, healthy control; B, high-risk population for type 2 diabetes; C, newly diagnosed type 2 diabetes; D, type 2 diabetes diagnosed for more than two years. The *y* axis is the relative concentration.

In this study, triglyceride levels showed changes, but no consistent trends were observed in high-risk or newly diagnosed T2D patients, suggesting diet and lifestyle may play a larger role. Clinically, total triglyceride levels showed a continuous increase in these groups. After two years of medication, triglyceride levels decreased, indicating that treatment or lifestyle changes can improve lipid profiles (Figure 13).

The changes in triglyceride (TG) levels observed in this study underscore the complex relationship between lipid metabolism and type 2 diabetes (T2D). The absence of consistent trends in TG levels among high-risk and newly diagnosed T2D patients suggests that diet and lifestyle significantly influence triglyceride metabolism.

The continuous increase in total triglyceride levels indicates a worsening lipid profile, contributing to insulin resistance and metabolic dysfunction [27]. Elevated triglyceride levels, particularly those enriched in short-chain saturated fatty acids, may disrupt insulin signaling and promote inflammation [30].

However, the decrease in triglyceride levels after two years of treatment highlights the effectiveness of pharmacological and lifestyle interventions in improving lipid profiles. This reduction is essential for lowering the risk of cardiovascular complications associated with T2D.

#### 4.1.6. Dynamic Change in Cholesterol Ester (CE) Metabolism

Cholesterol is a crucial metabolite and precursor for various bioactive substances [32]. It exists in the blood primarily as high-density lipoprotein cholesterol (HDL-C), low-density lipoprotein cholesterol (LDL-C), and very low-density lipoprotein cholesterol (VLDL-C). Most blood cholesterol is in the form of cholesterol esters (CE) bound to fatty acids, with less than 10% in a free state. Low HDL-C and high LDL-C levels are independent risk factors for coronary heart disease in type 2 diabetes (T2D) patients [33].

**Figure 14:**
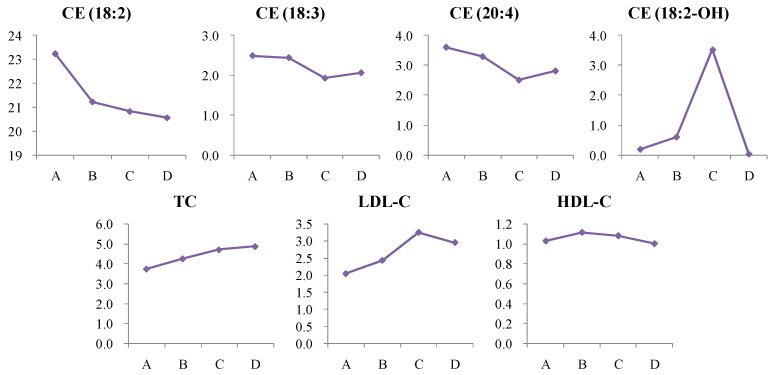
Change trends of cholesteryl ester (CE) in different groups. The *x* axis is the experimental group. A, healthy control; B, high-risk population for type 2 diabetes; C, newly diagnosed type 2 diabetes; D, type 2 diabetes diagnosed for more than two years. The *y* axis is the relative concentration.

In this study, total cholesterol (TC) and LDL-C levels were significantly elevated in newly diagnosed T2D patients and high-risk groups, with no significant change in HDL-C levels. Cholesterol esters containing polyunsaturated fatty acids—CE (18:2), CE (18:3), and CE (20:4)—were significantly reduced in newly diagnosed T2D patients, with CE (18:2) also decreasing in high-risk individuals and further declining in patients after more than two years’ treatment (Figure 14). These CEs are likely negatively correlated with T2D progression, potentially affecting insulin sensitivity and lipid metabolism. The elevation of CE (18:2-OH), a hydroxylated form of linoleic acid, in both newly diagnosed T2D patients and high-risk groups suggests increased oxidative stress in diabetes (Figure 14). Hydroxyl linoleic acid acts as an agonist of peroxisome proliferator-activated receptor γ (PPARγ), which is implicated in inflammation, atherosclerosis, insulin resistance, and glucose metabolism [34]. The reduction in polyunsaturated fatty acid-containing CEs may disrupt cellular membrane integrity and signaling, further contributing to insulin resistance and metabolic dysfunction. Elevated CE (18:2-OH) may also exacerbate inflammatory processes, increasing cardiovascular risk and promoting atherosclerosis [35].

The restoration of CE levels to normal levels after more than two years of treatment suggests the effectiveness of therapeutic interventions in re-establishing CE homeostasis, which may contribute to improved metabolic health. Nevertheless, additional research is necessary to elucidate the mechanisms behind these changes and their implications for treatment approaches.

### 4.2. Dynamic Lipid Biomarkers for T2D

To assess the diagnostic performance of differential metabolites and clinical markers for newly diagnosed type 2 diabetes (T2D), a receiver operating characteristic (ROC) curve analysis was conducted. The area under the curve (AUC), sensitivity, and specificity were calculated for each biomarker. Biomarkers with an AUC ≥ 0.7 were considered to have moderate to high diagnostic accuracy [36]. As shown in Table 4, among the clinical markers, fasting blood glucose (FPG) and glycated hemoglobin (HbA1c) demonstrated strong diagnostic potential. Additionally, total triglyceride (TG), low-density lipoprotein (LDL-C), and body mass index (BMI) exhibited some diagnostic value for T2D. Furthermore, 20 of the differential metabolites displayed diagnostic potential for T2D (AUC ≥ 0.7), with 8 of these metabolites exhibiting high diagnostic accuracy (AUC ≥ 0.9).

**Figure 15:**
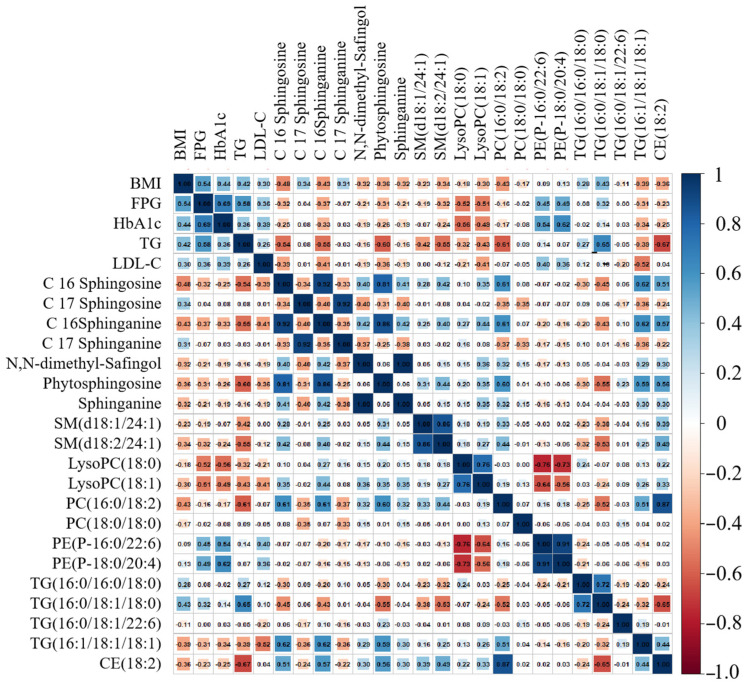
Correlation analysis of key clinical status and significantly changed lipids.

A correlation analysis revealed diverse associations between the 20 differential metabolites and clinical markers (Figure 15). Several lipid species were negatively correlated with triglycerides (TG): C16 Sphingosine, C16 Sphinganine, Phytosphingosine, SM(d18:2/24:1), PC(16:0/18:2), and CE(18:2) (r < −0.5). Conversely, TG(16:0/18:1/18:0) was positively correlated with TG (r > 0.5). LysoPC(18:0) was negatively correlated with HbA1c (r < −0.5), while PE(P-16:0/22:6) and PE(P-18:0/20:4) were positively correlated with HbA1c (r > 0.5). Additionally, LysoPC(18:0) and LysoPC(18:1) were negatively correlated with FPG, and TG(16:1/18:1/18:1) was negatively correlated with LDL-C (r < −0.5). These findings suggest a complex interplay between lipid metabolism and T2D. The negative correlation between certain lipid species and TG indicates a potential protective role against dyslipidemia. Conversely, the positive correlation between TG(16:0/18:1/18:0) and TG highlights its potential contribution to hypertriglyceridemia. Furthermore, the associations between specific lipids and glycemic control, as evidenced by the correlations with HbA1c and FPG, suggest that these lipid species may play a role in insulin resistance and glucose metabolism. 

While these findings highlight the complex interplay between lipid metabolism and T2D, it is important to note that 8 of the 20 differential metabolites did not show strong correlations with the clinical markers (r < 0.5 or r > −0.5). This underscores the multifaceted nature of T2D and the need for further investigation to fully understand the underlying metabolic dysregulation.

Among the 20 differential metabolites, 13 metabolites (Table 2, Table 3 and Table 4) demonstrated a consistent pattern of gradual increase or decrease in both high-risk individuals and newly diagnosed type 2 diabetes patients, indicating that these may serve as potential biomarkers for monitoring the progression of type 2 diabetes. 

Specifically, TG (16:0/18:1/18:0), TG (16:0/18:1/18:0), and CE (18:2-OH) exhibited a gradual increase in high-risk groups and newly diagnosed patients. The other 10 metabolites, including C16 sphingosine, C16 dihydrosphingosine, phytosphingosine, sphingosine, SM (d18:1/24:1), SM (d18:2/24:1), LysoPC (18:0), LysoPC (18:1), PC (18:0/18:0), and TG (16:0/16:0/18:0), showed a gradual decrease in the high-risk population and newly diagnosed patients. 

Notably, Sphinganine, CE(18:2-OH), PC(18:0/18:0), C16 Sphingosine, and C16 Sphinganine demonstrated more pronounced changes than traditional markers like FPG and HbA1c in the high-risk groups, suggesting their potential as more sensitive early biomarkers for T2D. Additionally, the persistence of altered LysoPC (18:0) and LysoPC (18:1) levels after prolonged treatment suggests their potential as biomarkers for insulin resistance.

## 5. Conclusions

This comprehensive lipidomics study, employing both untargeted and targeted approaches, reveals dynamic alterations in lipid metabolism across various stages of type 2 diabetes mellitus (T2DM). By analyzing serum samples from a diverse cohort, we identified significant changes in 44 lipid metabolites in newly diagnosed patients and 29 in high-risk individuals, compared with the healthy controls. Key metabolic pathways, including sphingomyelin, phosphatidylcholine, and sterol ester metabolism, were disrupted, highlighting the critical role of insulin resistance and oxidative stress in T2DM progression. Medications or lifestyle adjustment might restore the altered lipid metabolism to some extent, as is seen from the data of the MTYT2D group.

Furthermore, 13 lipid metabolites demonstrated diagnostic potential, exhibiting consistent trends of increase or decrease with disease progression. Our findings underscore the importance of lipid metabolism in T2D development and identify promising lipid biomarkers for early diagnosis, disease monitoring, and the evaluation of therapeutic interventions. These results contribute to paving the way for novel therapeutic strategies targeting lipid metabolism to address the complexities of T2DM. 

There are also some limitations in this study. For example, the cross-sectional nature of this study limits the ability to infer causality between altered lipid metabolism and T2D progression. Also, functional assays to test the impact of lipid changes on insulin resistance or related metabolic processes are lacking. Case–control study or cohort study of larger populations might be performed in the future to infer causality between altered lipid metabolism and T2D progression and validate the diagnosability of the lipid biomarkers found in this study.

## Figures and Tables

**Table 1:** Clinical characteristics of participants.

	Control Group	HR Group	NDT2D Group	MTYT2D Group
No. of subjects	40	40	39	36
Age (year)	53.7 ± 7.0	53.4 ± 6.1	50.2 ± 8.0	51.7 ± 10.8
BMI (kg/m^2^)	22.4 ± 0.6	26.4 ± 0.9 ***	25.6 ± 5.3 ***	26.5 ± 3.9 ***
FPG (mmol/L)	4.87 ± 0.65	5.23 ± 0.98 *	10.3 ± 3.59 ***^###^	8.24 ± 2.41 ***^&^
HbA1c (%)	5.65 ± 0.22	6.13 ± 0.20 ***	8.16 ± 2.59 ***^###^	7.88 ± 1.85 ***
TC (mmol/L)	3.73 ± 0.91	4.23 ± 0.87 **	4.74 ± 1.11 ***	4.87 ± 1.03 ***
TG (mmol/L)	1.19 ± 0.59	1.40 ± 0.59	2.45 ± 2.02 ***^##^	2.16 ± 2.08 **
LDL-C (mmol/L)	2.05 ± 0.68	2.41 ± 0.63 *	3.28 ± 2.40 ***^#^	2.95 ± 0.95 **
HDL-C (mmol/L)	1.03 ± 0.26	1.11 ± 0.63	1.18 ± 0.24	1.00 ± 0.26

Note: BMI, body mass index; FPG, fasting plasma glucose; HbA1c, glycated hemoglobin; TC, total cholesterol; TG, hlyceryl tridodecanoate; LDL-C, low-density lipoprotein cholesterol; HDL-C, high-density lipoprotein cholesterol. Data are shown as mean ± standard deviations. * *p* < 0.05, ** *p* < 0.01, *** *p* < 0.001, compared with the control group; ^#^ *p* < 0.05, ^##^ *p* < 0.01, ^###^ *p* < 0.001, compared with the HR group; and ^&^ *p* < 0.05, compared with the NDT2D group.

**Table 2:** The discriminated metabolites between control and HR groups obtained via the LC-(±)MS/MS analysis.

RT	*m/z*	Adduct Ion	Elemental Composition	Metabolite Identification ^a,b^	Category	*p* Value	Fold Change (HR/Control)	VIP
1.41	318.2993	[M+H]^+^	C_18_H_39_NO_3_	Phytosphingosine ^b^	Sphingolipids	2.46 × 10^−3^	0.85	2.82
1.43	274.2732	[M+H]^+^	C_16_H_35_NO_2_	C16 Sphinganine ^b^		1.66 × 10^−5^	0.75	15.0
1.60	288.2889	[M+H]^+^	C_17_H_37_NO_2_	C17 Sphinganine ^b^		1.15 × 10^−11^	4.62	18.9
1.64	272.2578	[M+H]^+^	C_16_H_33_NO_2_	C16 Sphingosine ^b^		3.19 × 10^−7^	0.70	1.30
1.86	302.3047	[M+H]^+^	C_18_H_39_NO_2_	Sphinganine ^b^		4.03 × 10^−5^	0.03	6.48
1.90	286.2734	[M+H]^+^	C_17_H_35_NO_2_	C17 Sphingosine ^b^		1.70 × 10^−10^	4.24	1.73
2.73	330.3358	[M+H]^+^	C_20_H_43_NO_2_	N,N-dimethyl-Safingol ^b^		4.89 × 10^−5^	0.05	4.29
15.44	855.6609	[M+FA−H]^−^	C_47_H_91_N_2_O_6_P	SM(d18:2/24:1) ^b^		3.20 × 10^−4^	0.73	2.48
15.48	811.6665	[M+H]^+^	C_47_H_91_N_2_O_6_P			4.29 × 10^−2^	0.89	1.39
17.00	857.6768	[M+FA−H]^−^	C_47_H_93_N_2_O_6_P	SM(d18:1/24:1) ^b^		2.64 × 10^−3^	0.78	2.42
3.77	566.3469	[M+FA−H]^−^	C_27_H_54_NO_9_P	LysoPC(18:1) ^a,b^	Lysophosphatidylcholine (LysoPC)	6.36 × 10^−4^	0.80	1.65
5.18	568.3625	[M+FA−H]^−^	C_26_H_54_NO_7_P	LysoPC(18:0) ^a,b^		2.33 × 10^−5^	0.89	2.46
3.25	496.3388	[M+H]^+^	C_24_H_50_NO_7_P	LysoPC(16:0) ^a^		9.75 × 10^−4^	0.71	2.31
14.05	808.5800	[M+Na]^+^	C_44_H_84_NO_8_P	PC(18:0/18:2) ^a,b^	Phosphatidylcholine (PC)	4.51 × 10^−4^	0.87	2.89
12.47	780.5487	[M+Na]^+^	C_42_H_80_NO_8_P	PC(16:0/18:2) ^a,b^		7.17 × 10^−7^	0.85	5.54
12.56	784.5827	[M+Na]^+^	C_42_H_84_NO_8_P	PC(16:0/18:0) ^b^		3.77 × 10^−3^	0.73	6.02
12.56	806.5648	[M+Na]^+^	C_44_H_82_NO_8_P	PC(18:1/18:2) ^b^		1.89 × 10^−3^	0.78	1.95
13.24	742.5729	[M+H]^+^	C_42_H_80_NO_7_P	PC(O-16:1/18:2) ^b^		4.40 × 10^−2^	0.85	1.18
13.22	838.5980	[M+FA−H]^−^	C_46_H_84_NO_7_P	PC(P-18:0/20:4)		4.55 × 10^−3^	0.73	1.01
13.74	880.6082	[M−H]^−^	C_49_H_88_NO_10_P	PC(18:0/22:5) ^b^		8.01 × 10^−4^	0.79	1.07
12.47	758.5677	[M+H]^+^	C_42_H_80_NO_8_P	PC(18:2/16:0)		9.74 × 10^−4^	0.88	11.7
13.26	878.5931	[M+FA−H]^−^	C_48_H_84_NO_8_P	PC(18:0/22:6) ^a^		4.20 × 10^−2^	0.85	1.20
13.62	734.5679	[M+H]^+^	C_40_H_80_NO_8_P	PC(16:0/16:0)		6.32 × 10^−3^	0.83	1.85
13.68	782.5648	[M+Na]^+^	C_42_H_82_NO_8_P	PC(16:0/18:1) ^a^		3.58 × 10^−3^	0.88	1.90
21.95	920.7677	[M+NH4]^+^	C_59_H_98_O_6_	TG(16:0/18:2/22:6) ^b^	Triglycerides (TG)	1.57 × 10^−2^	1.46	2.72
22.42	879.7385	[M+Na]^+^	C_55_H_100_O_6_	TG(16:1/18:1/18:1) ^b^		1.39 × 10^−6^	0.87	3.31
21.35	712.6435	[M+NH4]^+^	C_43_H_82_O_6_	TG(12:0/14:0/14:0)		7.32 × 10^−9^	0.59	1.76
21.62	918.7527	[M+NH4]^+^	C_59_H_96_O_6_	TG(18:2/20:5/18:2)		1.02 × 10^−3^	2.03	1.46
21.80	740.6746	[M+NH4]^+^	C_45_H_86_O_6_	TG(12:0/14:0/16:0)		3.429 × 10^−9^	0.68	1.54
22.80	671.5718	[M+Na]^+^	C_45_H_76_O_2_	CE(18:2) ^b^	Cholesterol ester (CE)	1.49 × 10^−2^	0.91	2.14

^a^ Metabolites confirmed by standard compounds; ^b^ metabolites distinguishing the control group from the HR group and simultaneously distinguishing the control group from the NDT2D group. Control: healthy individuals; HR: high-risk individuals for type 2 diabetes; NDT2D: newly diagnosed type 2 diabetes patients.

**Table 3:** The discriminated metabolites between the control and NDT2D groups obtained via the LC-(±)MS/MS analysis.

RT	*m/z*	Adduct Ion	Elemental Composition	Metabolite Identification ^a,b^	Category	*p* Value	Fold Change (NDT2D/Control)	VIP
1.41	318.2993	[M+H]^+^	C_18_H_39_NO_3_	Phytosphingosine ^b^	Sphingolipids	1.58 × 10^−9^	0.64	4.99
1.43	274.2732	[M+H]^+^	C_16_H_35_NO_2_	C16 Sphinganine ^b^		3.25 × 10^−12^	0.53	21.6
1.60	288.2889	[M+H]^+^	C_17_H_37_NO_2_	C17 Sphinganine ^b^		5.49 × 10^−16^	4.22	16.3
1.64	272.2578	[M+H]^+^	C_16_H_33_NO_2_	C16 Sphingosine ^b^		1.81 × 10^−12^	0.52	1.68
1.86	302.3047	[M+H]^+^	C_18_H_39_NO_2_	Sphinganine ^b^		4.12 × 10^−5^	0.02	5.56
1.90	286.2734	[M+H]^+^	C_17_H_35_NO_2_	C17 Sphingosine ^b^		1.12 × 10^−24^	4.53	1.79
2.73	330.3358	[M+H]^+^	C_20_H_43_NO_2_	N,N-dimethyl-Safingol ^b^		4.35 × 10^−5^	0.03	3.71
15.48	811.6665	[M+H]^+^	C_47_H_91_N_2_O_6_P	SM(d18:2/24:1) ^b^		4.47 × 10^−5^	0.75	2.73
15.44	855.6609	[M+FA−H]^−^	C_47_H_91_N_2_O_6_P			3.88 × 10^−3^	0.76	4.30
17.00	857.6768	[M+FA−H]^−^	C_47_H_93_N_2_O_6_P	SM(d18:1/24:1) ^b^		2.28 × 10^−2^	0.81	3.90
17.05	813.6825	[M+H]^+^	C_47_H_93_N_2_O_6_P			1.21 × 10^−3^	0.79	2.46
12.06	703.5733	[M+H]^+^	C_39_H_79_N_2_O_6_P	SM(d18:1/16:0) ^a^		2.98 × 10^−2^	0.88	2.98
19.92	692.6211	[M+FA−H]^−^	C_42_H_81_NO_3_	Cer(d18:1/24:1)		5.31 × 10^−3^	1.48	1.19
3.79	522.3545	[M+H]^+^	C_26_H_52_NO_7_P	LysoPC(18:1) ^a,b^	Lysophosphatidylcholine (LysoPC)	4.03 × 10^−4^	0.81	1.78
3.77	566.3469	[M+FA−H]^−^	C_26_H_52_NO_7_P			1.04 × 10^−2^	0.84	2.02
5.21	524.3701	[M+H]^+^	C_26_H_54_NO_7_P	LysoPC(18:0) ^a,b^		9.97 × 10^−5^	0.81	3.16
5.18	568.3625	[M+FA−H]^−^	C_26_H_54_NO_7_P			9.63 × 10^−8^	0.85	4.79
2.77	520.3389	[M+H]^+^	C_26_H_50_NO_7_P	LysoPC(18:2)		3.71 × 10^−2^	0.84	1.69
12.47	780.5487	[M+Na]^+^	C_42_H_80_NO_8_P	PC(16:0/18:2) ^a,b^	Phosphatidylcholine (PC)	9.05 × 10^−4^	0.89	3.31
12.56	784.5827	[M+Na]^+^	C_42_H_84_NO_8_P	PC(16:0/18:0) ^b^		3.43 × 10^−2^	0.81	3.99
12.56	806.5648	[M+Na]^+^	C_44_H_82_NO_8_P	PC(18:1/18:2) ^b^		1.08 × 10^−2^	0.83	1.44
13.24	742.5729	[M+H]^+^	C_42_H_80_NO_7_P	PC(O-16:1/18:2) ^b^		3.24 × 10^−2^	0.84	1.10
13.75	832.5801	[M+Na]^+^	C_46_H_84_NO_8_P	PC(18:0/20:4) ^a^		6.57 × 10^−3^	0.84	1.58
14.05	808.5800	[M+Na]^+^	C_44_H_84_NO_8_P	PC(18:0/18:2) ^a,b^		4.36 × 10^−3^	0.90	2.31
13.74	880.6082	[M−H]^−^	C_49_H_88_NO_10_P	PC(18:0/22:5) ^b^		2.04 × 10^−3^	0.82	1.46
14.01	812.6125	[M+Na]^+^	C_44_H_88_NO_8_P	PC(18:0/18:0)		7.03 × 10^−7^	0.36	1.83
14.43	856.6086	[M+FA−H]^−^	C_46_H_86_NO_8_P	PC(18:0/20:3)		1.02 × 10^−2^	0.84	2.53
15.37	788.6140	[M+H]^+^	C_44_H_86_NO_8_P	PC(16:0/20:1)		2.21 × 10^−3^	0.85	2.65
12.91	746.5143	[M−H]^−^	C_43_H_74_NO_7_P	PE(P-16:0/22:6)	Phosphatidylethanolamine (PE)	2.69 × 10^−3^	1.27	1.14
13.39	722.5144	[M−H]^−^	C_41_H_74_NO_7_P	PE(P-16:0/20:4)		1.72 × 10^−10^	1.78	1.52
15.05	750.5456	[M−H]^−^	C_43_H_78_NO_7_P	PE(P-18:0/20:4)		4.72 × 10^−2^	1.22	1.62
21.95	920.7677	[M+NH4]^+^	C_59_H_98_O_6_	TG(16:0/18:2/22:6) ^b^	Triglycerides (TG)	4.14 × 10^−2^	1.30	1.50
22.42	879.7385	[M+Na]^+^	C_55_H_100_O_6_	TG(16:1/18:1/18:1) ^b^		1.58 × 10^−4^	0.89	2.15
21.65	894.7523	[M+NH4]^+^	C_57_H_96_O_6_	TG(18:2/18:2/18:3)		4.02 × 10^−2^	0.69	1.77
22.08	927.7384	[M+Na]^+^	C_59_H_100_O_6_	TG(16:0/18:1/22:6)		1.65 × 10^−4^	0.65	1.76
22.19	898.7833	[M+NH4]^+^	C_57_H_100_O_6_	TG(16:0/18:1/20:4)		3.11 × 10^−2^	0.85	4.34
22.41	900.7985	[M+NH4]^+^	C_57_H_102_O_6_	TG(18:1/18:1/18:2)		2.89 × 10^−2^	0.87	4.32
22.65	850.7839	[M+NH4]^+^	C_53_H_100_O_6_	TG(16:0/16:0/18:1)		1.37 × 10^−2^	1.23	4.24
22.88	904.8303	[M+NH4]^+^	C_57_H_106_O_6_	TG(18:1/18:1/18:0)		4.09 × 10^−2^	1.33	3.06
22.90	878.8150	[M+NH4]^+^	C_55_H_104_O_6_	TG(16:0/18:1/18:0)		2.05 × 10^−3^	1.68	5.31
22.92	852.7994	[M+NH4]^+^	C_53_H_102_O_6_	TG(16:0/16:0/18:0)		4.15 × 10^−5^	1.46	1.41
23.17	906.8463	[M+NH4]^+^	C_57_H_108_O_6_	TG(18:1/18:0/18:0)		2.65 × 10^−2^	1.72	1.60
22.63	876.7993	[M+NH4]^+^	C_55_H_102_O_6_	TG(16:0/18:1/18:1)		7.63 × 10^−3^	1.22	7.80
22.80	671.5718	[M+Na]^+^	C_45_H_76_O_2_	CE(18:2) ^b^	Cholesterol ester (CE)	2.81 × 10^−2^	0.90	1.84
20.71	687.5680	[M+Na]^+^	C_45_H_76_O_3_	CE(18:2-OH)		1.08 × 10^−5^	18.2	3.12
22.53	664.6013	[M+NH4]^+^	C_45_H_74_O_2_	CE(18:3)		3.45 × 10^−2^	0.78	1.03
22.59	695.5720	[M+Na]^+^	C_47_H_76_O_2_	CE(20:4)		3.99 × 10^−2^	0.70	1.14
17.43	617.5102	[M+Na]^+^	C_37_H_7_0O_5_	DG(16:0/18:1)	Diacylglycerol	2.83 × 10^−3^	1.44	1.24

^a^ Metabolites confirmed by standard compounds; ^b^ metabolites distinguishing the control group from the NDT2D group and simultaneously distinguishing the control group from the HR group. Control: healthy individuals; HR: high-risk individuals for type 2 diabetes; NDT2D: newly diagnosed type 2 diabetes patients.

**Table 4:** Discriminated metabolites with moderate to high diagnostic power (AUC ≥ 0.7) for newly diagnosed type 2 diabetes.

Discriminated Metabolites	AUC	Sensitivity	Specificity	Change Trend *
FPG	1.000	1.000	1.000	↑
HbA1c	0.920	0.920	1.000	↑
TG	0.893	0.800	0.925	↑
LDL-C	0.845	0.800	0.800	↑
BMI	0.833	0.800	1.000	↑
C17 Sphingosine	1.000	1.000	1.000	↑
Sphinganine	0.985	1.000	0.949	↓
C16 Sphingosine	0.960	0.950	0.949	↓
C16 Sphinganine	0.956	0.950	0.872	↓
N,N-dimethyl-Safingol	0.936	0.875	0.821	↓
C17 Sphinganine	0.935	1.000	0.850	↑
Phytosphingosine	0.917	0.875	0.821	↓
PE(P-16:0/22:6)	0.900	1.000	0.825	↑
TG(16:0/16:0/18:0)	0.873	0.800	0.900	↑
PC(18:0/18:0)	0.798	0.750	0.769	↓
PE(P-18:0/20:4)	0.785	0.800	0.775	↑
SM(d18:2/24:1)	0.764	0.725	0.692	↓
LysoPC(18:1)	0.752	0.875	0.615	↓
TG(16:0/18:1/18:0)	0.745	1.000	0.525	↑
TG(16:0/18:1/22:6)	0.736	0.650	0.692	↓
TG(16:1/18:1/18:1)	0.731	0.725	0.667	↓
LysoPC(18:0)	0.726	0.525	0.872	↓
CE(18:2-OH)	0.724	0.641	0.700	↑
SM(d18:1/24:1)	0.720	0.725	0.692	↓
PC(16:0/18:2)	0.700	0.625	0.667	↓

* Note: relative concentration in the NDT2D group down-regulated (↓) or up-regulated (↑) compared with the Control group.

## Data Availability

The raw data supporting the conclusions of this article will be made available by the authors on request.

## Supplementary Materials

The following supporting information can be downloaded at: https://www.mdpi.com/article/10.3390/metabo14110610/s1, Figure S1: The retention time deviation profiles deriving from LC-(±)ESIMS. A positive deviation indicates that the sample was eluting after the median retention time, and a negative deviation indicates that the sample was eluting before the median retention time; Figure S2. Line plots of quality control (QC) samples for LC-(+)ESIMS analysis generated by PCA using component 1 (A) and 2 (B). Peak area deviation could be evaluated by distribution of the runs. *X*-axis: run order; *Y*-axis: standard deviation; Figure S3. Line plots of quality control (QC) samples for LC-(−)ESIMS analysis generated by PCA using component 1 (A) and 2 (B). Peak area deviation could be evaluated by distribution of the runs. *X*-axis: run order; *Y*-axis: standard deviation; Figure S4. OPLS-DA score plots based on (A) LC-(+)ESIMS data (B) LC-(−) ESIMS data from the control and NDT2D groups (: control group, : NDT2D group); Figure S5. PLS-DA validation plots of 100 random permutations based on the (A) LC-(+)ESIMS and (B) LC-(−)ESIMS data from the control and NDT2D groups; Figure S6. OPLS-DA score plots based on (A) LC-(+)ESIMS data (B) LC-(−) ESIMS data from control group and MTYT2D group (: control group, : NDT2D group); Figure S7. PLS-DA validation plots of 100 random permutations based on the (A) LC-(+)ESIMS and (B) LC-(−)ESIMS data from the control group and MTYT2D group; Table S1: Information of 17 typical standards from six lipids categories.; Table S2. Summary of discriminated metabolites between the control and HR groups via the LC-(+)ESIMS analysis; Table S3. Summary of discriminated metabolites between the control and NDT2TD groups via the LC-(+)ESIMS analysis; Table S4. Summary of discriminated metabolites between the control and HR groups via the LC-(−)ESIMS analysis; Table S5: Summary of discriminated metabolites between the control and NDT2TD groups via the LC-(−)ESIMS analysis; Table S6. The parameters of the LC-MRM-MS-based targeted metabolomic analysis in positive ion mode; Table S7: The parameters of the LC-MRM-MS-based targeted metabolomic analysis in negative ion mode.

## References

[1] Arroyo M.N., Green J.A., Cnop M., Igoillo-Esteve M. (2021). tRNA Biology in the Pathogenesis of Diabetes: Role of Genetic and Environmental Factors. Int. J. Mol. Sci..

[2] Chaki J., Ganesh S.T., Cidham S.K., Theertan S.A. (2020). Machine Learning and Artificial Intelligence based Diabetes Mellitus Detection and Self-Management: A Systematic Review. J. King Saud Univ.—Comput. Inf. Sci..

[3] Jiang S., Young J.L., Wang K., Qian Y., Cai L. (2020). Diabeticinduced alterations in hepatic glucose and lipid metabolism: The role of type 1 and type 2 diabetes mellitus (Review). Mol. Med. Rep..

[4] Frayn K.N. (1993). Insulin resistance and lipid metabolism. Curr. Opin. Lipidol..

[5] Chen H., Wu J., Lyu R. (2024). Expressions of glycemic parameters, lipid profile, and thyroid hormone in patients with type 2 diabetes mellitus and their correlation. Immun. Inflamm. Dis..

[6] Tautenhahn R., Patti G.J., Rinehart D., Siuzdak G. (2012). XCMS Online: A Web-Based Platform to Process Untargeted Metabolomic Data. Anal. Chem..

[7] Kuhl C., Tautenhahn R., Bottcher C., Larson T.R., Neumann S. (2012). CAMERA: An integrated strategy for compound spectra extraction and annotation of liquid chromatography/mass spectrometry data sets. Anal. Chem..

[8] Ihaka R., Gentleman R. (1996). R: A Language for Data Analysis and Graphics. J. Comput. Graph. Stat..

[9] Kind T., Fiehn O. (2007). Seven Golden Rules for heuristic filtering of molecular formulas obtained by accurate mass spectrometry. BMC Bioinform..

[10] Green C.D., Maceyka M., Cowart L.A., Spiegel S. (2021). Sphingolipids in metabolic disease: The good, the bad, and the unknown. Cell Metab..

[11] Chen Q., Wang W., Xia M.-F., Liu Y.-L., Bian H., Yu C., Li X.-Y., Vadas M.A., Gao X., Lin H.-D. (2021). Identification of circulating sphingosine kinase-related metabolites for prediction of type 2 diabetes. J. Transl. Med..

[12] Morita Y., Kurano M., Sakai E., Nishikawa T., Nishikawa M., Sawabe M., Aoki J., Yatomi Y. (2020). Analysis of urinary sphingolipids using liquid chromatography-tandem mass spectrometry in diabetic nephropathy. J. Diabetes Investig..

[13] Xia Q.-S., Lu F.-E., Wu F., Huang Z.-Y., Dong H. (2020). New role for ceramide in hypoxia and insulin resistance. World J. Gastroenterol..

[14] Summers S.A. (2006). Ceramides in insulin resistance and lipotoxicity. Prog. Lipid Res..

[15] Straczkowski M., Kowalska I., Baranowski M., Nikolajuk A., Otziomek E., Zabielski P., Adamska A., Blachnio A., Gorski J., Gorska M. (2007). Increased skeletal muscle ceramide level in men at risk of developing type 2 diabetes. Diabetologia.

[16] Law S.H., Chan M.L., Marathe G.K., Parveen F., Ke L.Y. (2019). An Updated Review of Lysophosphatidylcholine Metabolism in Human Diseases. Int. J. Mol. Sci..

[17] Susanne H., Marcus F., Andrea B., Gerhard L., Alexander S., Stefan W., Gerd S., Ayyalasomayajula V. (2014). Alterations of Plasma Lysophosphatidylcholine Species in Obesity and Weight Loss. PLoS ONE.

[18] Auguet T., Bertran L., Capellades J., Abelló S., Aguilar C., Sabench F., del Castillo D., Correig X., Yanes O., Richart C. (2023). LC/MS-Based Untargeted Metabolomics Analysis in Women with Morbid Obesity and Associated Type 2 Diabetes Mellitus. Int. J. Mol. Sci..

[19] Yea K., Kim J., Lim S., Kwon T., Park H.S., Park K.S., Suh P.-G., Ryu S.H. (2009). Lysophosphatidylserine regulates blood glucose by enhancing glucose transport in myotubes and adipocytes. Biochem. Biophys. Res. Commun..

[20] Parmryd I. (2023). Barrier and signal transduction functions could explain the lipid asymmetry of the plasma membrane. BioEssays.

[21] Sawada N., Obama T., Mizuno M., Fukuhara K., Iwamoto S., Aiuchi T., Makiyama T., Itabe H. (2020). Transfer and Enzyme-Mediated Metabolism of Oxidized Phosphatidylcholine and Lysophosphatidylcholine between Low- and High-Density Lipoproteins. Antioxidants.

[22] Raubenheimer P.J., Nyirenda M.J., Walker B.R. (2006). A choline-deficient diet exacerbates fatty liver but attenuates insulin resistance and glucose intolerance in mice fed a high-fat diet. Diabetes.

[23] Loomba R., Friedman S.L., Shulman G.I. (2021). Mechanisms and disease consequences of nonalcoholic fatty liver disease. Cell.

[24] Chakrabarti A. (2021). Phospholipid Asymmetry in Biological Membranes: Is the Role of Phosphatidylethanolamine Underappreciated?. J. Membr. Biol..

[25] Lee H.C., Cheng W.C., Ma W.L., Lin Y.H., Shin S.J., Lin Y.H. (2023). Association of lipid composition and unsaturated fatty acids of VLDL with atrial remodeling in metabolic syndrome. Sci. Rep..

[26] Xu H., Li W., Huang L., He X., Xu B., He X., Chen W., Wang Y., Xu W., Wang S. (2023). Phosphoethanolamine cytidylyltransferase ameliorates mitochondrial function and apoptosis in hepatocytes in T2DM in vitro. J. Lipid Res..

[27] Chamroonkiadtikun P., Ananchaisarp T., Wanichanon W. (2019). The triglyceride-glucose index, a predictor of type 2 diabetes development: A retrospective cohort study. Prim. Care Diabetes.

[28] Wang Y. (2021). Higher fasting triglyceride predicts higher risks of diabetes mortality in US adults. Lipids Health Dis..

[29] Rhee E.P., Cheng S., Larson M.G., Walford G.A., Gerszten R.E. (2011). Lipid profiling identifies a triacylglycerol signature of insulin resistance and improves diabetes prediction in humans. J. Clin. Investig..

[30] Sowah S.A., Hirche F., Milanese A., Johnson T.S., Grafetstätter M., Schübel R., Kirsten R., Ulrich C.M., Kaaks R., Zeller G. (2020). Changes in plasma short-chain fatty acid levels after dietary weight loss among overweight and obese adults over 50 weeks. Nutrients.

[31] Rooney M.R., Rawlings A.M., Pankow J.S., Tcheugui J.B.E., Coresh J., Sharrett A.R., Selvin E. (2021). Risk of progression to diabetes among older adults with prediabetes. JAMA Intern. Med..

[32] Sekhar M.S., Marupuru S., Reddy B.S., Kurian S.J., Rao M. (2020). Physiological Role of Cholesterol in Human Body. Dietary Sugar, Salt and Fat in Human Health.

[33] Zhao X., Wang D., Qin L. (2021). Lipid profile and prognosis in patients with coronary heart disease: A meta-analysis of prospective cohort studies. BMC Cardiovasc. Disord..

[34] Mirza A.Z., Althagafi I.I., Shamshad H. (2019). Role of PPAR receptor in different diseases and their ligands: Physiological importance and clinical implications. Eur. J. Med. Chem..

[35] Lozhkina N., Gushchina O., Basov N., Gaisler E., Rogachev A., Sotnikova Y.S., Patrushev Y.V., Pokrovsky A. (2024). Ceramides As Potential New Predictors of the Severity of Acute Coronary Syndrome in Conjunction with SARS-CoV-2 Infection. Acta Naturae.

[36] Janssens A.C.J., Martens F.K. (2020). Reflection on modern methods: Revisiting the area under the ROC Curve. Int. J. Epidemiol..

