# Supplementary material for: Untargeted and Targeted Lipidomics Unveil Dynamic Lipid Metabolism Alterations in Type 2 Diabetes
Source: Metabolites. 2024 Nov 10;14(11):610. doi: 10.3390/metabo14110610 (PMC11596168; doi:10.3390/metabo14110610)
Supplement: Supplementary file 1 [file metabolites-14-00610-s001.zip › metabolites-3248121-supplementary.pdf]

Supplementary Materials

Contents

1. Figures

The following supporting information can be downloaded at: www.mdpi.com/xxx/s1,

Figure S1: The retention time deviation profiles deriving from LC-(±)ESIMS. A positive deviation indicates that the sample was eluting after the median retention time, and a negative deviation indicates that the samples was eluting before the median retention time;

Figure S2. Line plots of quality control (QC) samples for LC-(+)ESIMS analysis generated by PCA using component 1（A） and 2（B）. Peak area deviation could be evaluated by distribution of the runs. X-axis: run order; Y-axis: standard deviation;

Figure S3. Line plots of quality control (QC) samples for LC-(-)ESIMS analysis generated by PCA using component 1（A） and 2（B）. Peak area deviation could be evaluated by distribution of the runs. X-axis: run order; Y-axis: standard deviation;

Figure S4. OPLS-DA score plots based on (A) LC-(+)ESIMS data (B) LC-(–) ESIMS data from the Control and NDT2D group (■: control group, ▲: NDT2D group);

Figure S5. PLS-DA validation plots of 100 random permutations based on the (A) LC-(+)ESIMS and (B) LC-(–)ESIMS data from the Control and NDT2D group;

Figure S6. OPLS-DA score plots based on (A) LC-(+)ESIMS data (B) LC-(－) ESIMS data from control group and MTYT2D group (: Control group, : MTYT2D group);

Figure S7. PLS-DA validation plots of 100 random permutations based on the (A) LC-(+)ESIMS and (B) LC-(－)ESIMS data from the Control group and MTYT2D group.

2. Tables

Table S1: Information of 17 typical standards from six lipids categories.;

Table S2. Summary of discriminated metabolites between the Control and HR group by LC-(+)ESIMS analysis;

Table S3. Summary of discriminated metabolites between the Control and NDT2TD group by LC-(+)ESIMS analysis.

Table S4. Summary of discriminated metabolites between the Control and HR group by LC-(－)ESIMS analysis.

Table S5: Summary of discriminated metabolites between the Control and NDT2TD group by LC-(－)ESIMS analysis;

Table S6. The parameters of the LC-MRM-MS-based targeted metabolomic analysis in positive ion mode;

Table S7: The parameters of the LC-MRM-MS-based targeted metabolomic analysis in negtive ion mode.

3. Program

XCMS program for peak recognition, peak filtering, peak alignment and CAMERA analysis: The XCMS software package peak recognition, peak filtering, peak alignment and CAMERA analysis program based on R language applied in the research of serum lipomics of type 2 diabetes (taking LC - (+) ESI-MS spectral data as an example).

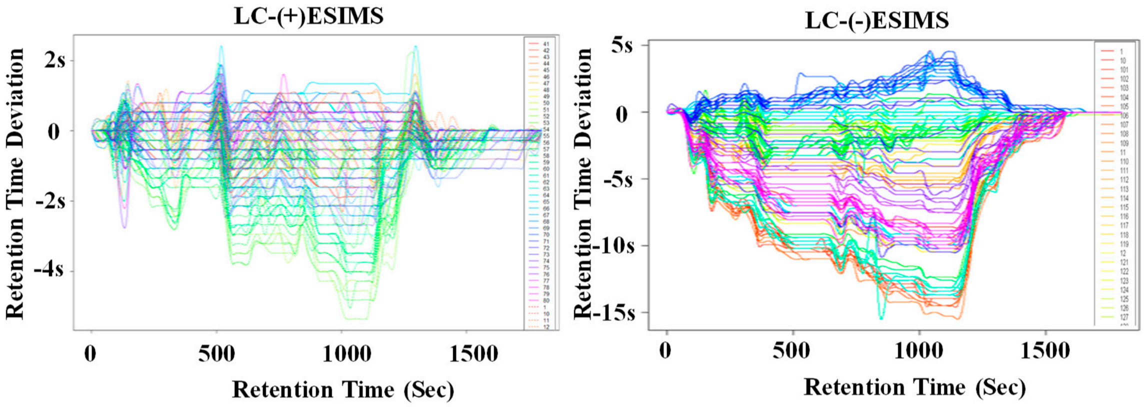

Figure S1. The retention time deviation profiles deriving from LC-(±)ESIMS. A positive deviation indicates that the sample was eluting after the median retention time, and a negative deviation indicates that the samples was eluting before the median retention time.

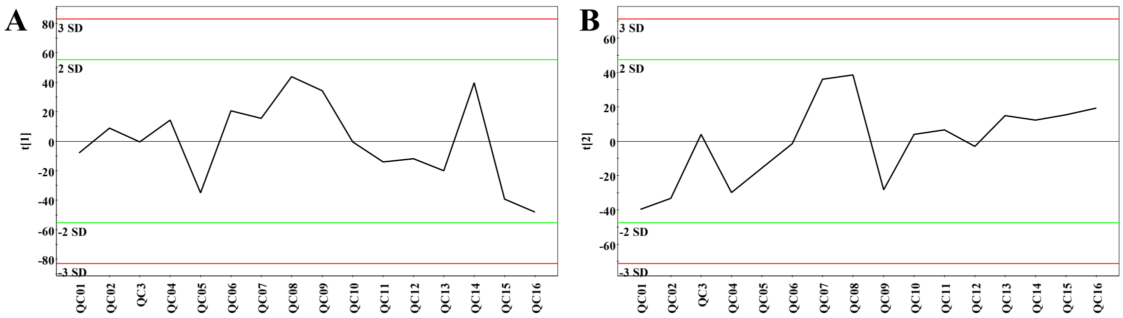

Figure S2. Line plots of quality control (QC) samples for LC-(+)ESIMS analysis generated by PCA using component 1（A）and 2（B）. Peak area deviation could be evaluated by distribution of the runs. X-axis: run order; Y-axis: standard deviation.

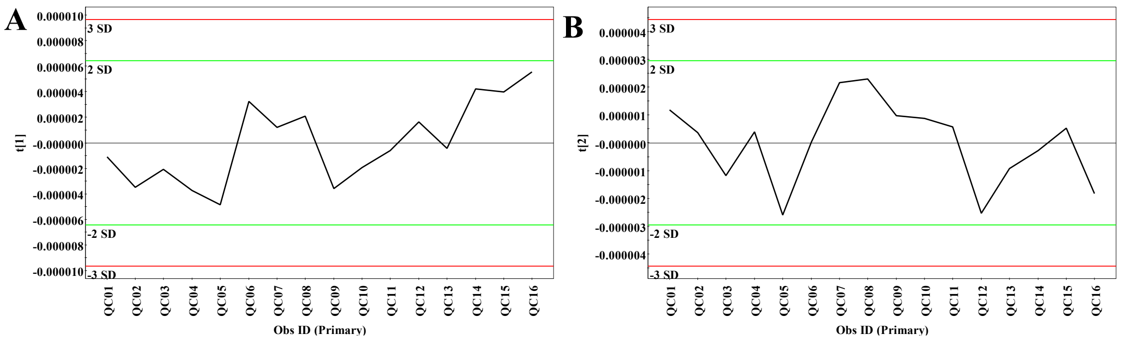

Figure S3. Line plots of quality control (QC) samples for LC-(-)ESIMS analysis generated by PCA using component 1（A）and 2（B）. Peak area deviation could be evaluated by distribution of the runs. X-axis: run order; Y-axis: standard deviation.

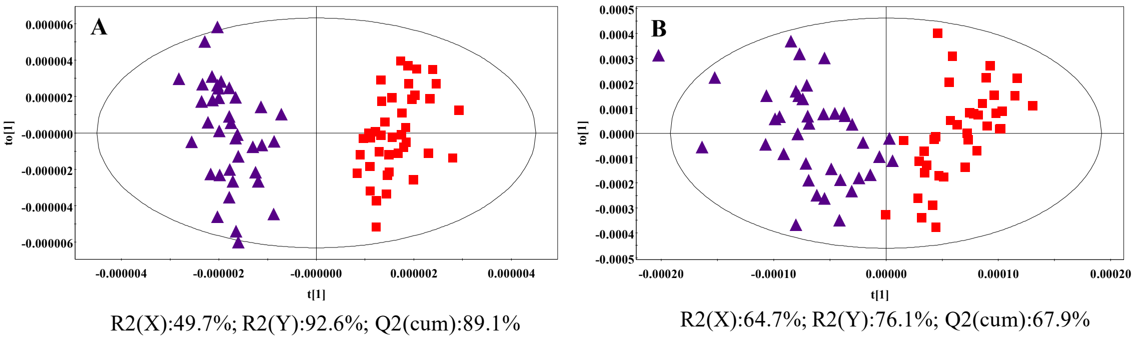

Figure S4. OPLS-DA score plots based on LC-(+)ESIMS data (A) (R2X:49.7%; R2(Y):92.6%; Q2(cum):89.1%) and LC-(–) ESIMS data (B) (R2X:64.7%; R2(Y):76.1%; Q2(cum):67.9%) from the Control and NDT2D group (■: control group, ▲: NDT2D group).

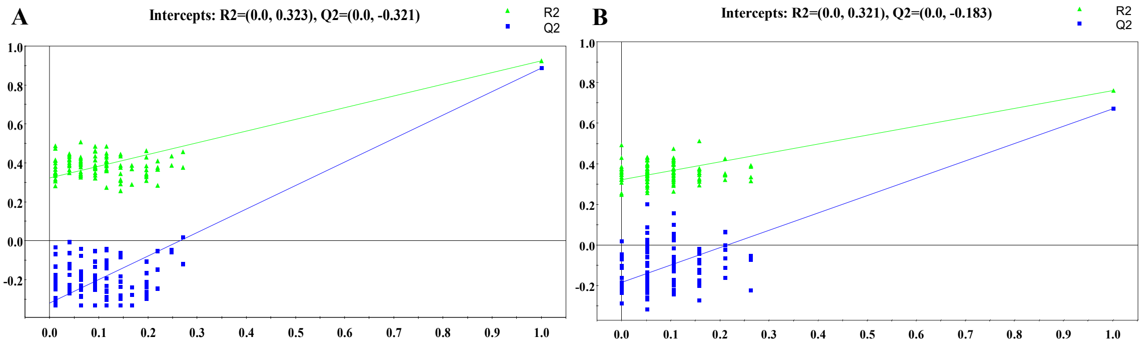

Figure S5. PLS-DA validation plots of 100 random permutations based on the LC-(+)ESIMS data (A) (R2=(0.0, 0.323), Q2=(0.0,-0.321))and LC-(–)ESIMS data (B) (R2=(0.0, 0.321), Q2=(0.0,-0.183)) from the Control and NDT2D group.

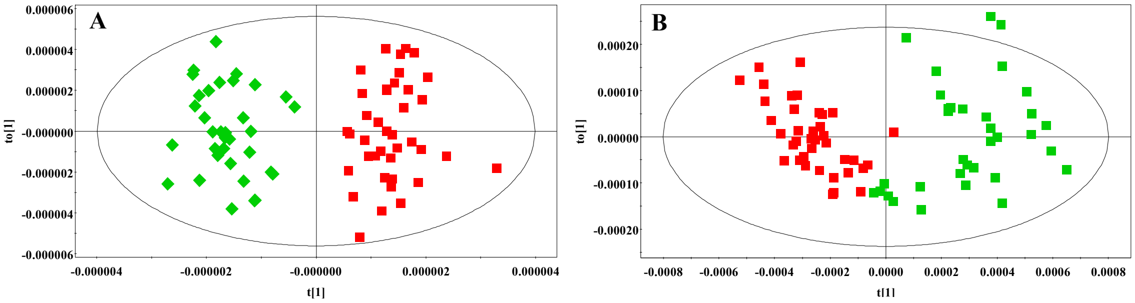

R2(X):45.1%; R2(Y):88.9%; Q2(cum):81.7% R2(X):82.5%; R2(Y):77.1%; Q2(cum):61.3%

Figure S6. OPLS-DA score plots based on LC-(+)ESIMS data (A) (R2X:45.1%; R2(Y):88.9%; Q2(cum):81.7%) and LC-(–) ESIMS data (B) (R2X:82.5%; R2(Y):77.1%; Q2(cum):61.3%) from control group and MTYT2D group (■: Control group, ■: MTYT2D group).

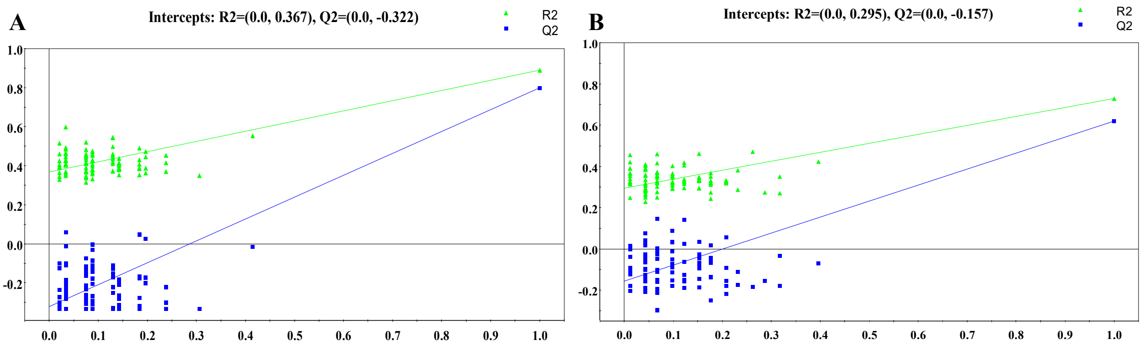

Figure S7. PLS-DA validation plots of 100 random permutations based on the LC-(+)ESIMS data (A) (R2=(0.0, 0.355), Q2=(0.0,-0.126)) and LC-(–)ESIMS data (B) (R2=(0.0, 0.355), Q2=(0.0,-0.126)) from the Control group and MTYT2D group.

Table S1 Information of 17 typical standards from six lipids categories.

**Lipid type and name**	**Abbreviation**	**Theoretical mass(Da)**	**Measured mass（Da）**	**Deviation（δ）**
Fatty acids				
Palmitic acid	FFA(16:0)	256.2402	256.2398	-1.6
Linoleic acid	FFA(18:2)	280.2402	280.2406	1.4
Glycerolipids				
1-Oleoyl-rac-glycerol	MG(18:1/0:0/0:0)	356.2927	356.292	-2
1-Stearoyl-rac-glycerol	MG(18:0/0:0/0:0)	358.3083	358.3078	-1.4
1,2-Dioleoyl-sn-glycerol	DG(18:1/18:1/0:0)	620.5380	620.5401	3.4
Glyceryltripalmitate	TG(16:0/16:0/16:0)	806.7363	806.7354	-1.1
Glyceryltrimyristate	TG(14:0/14:0/14:0)	722.6424	722.6435	1.5
Sphingolipids				
Sphinganine	SA	301.5078	301.5083	1.7
N-Palmitoyl-D-sphingomyelin	SM(d18:1/16:0)	702.5676	702.5671	-0.7
Glycerophospholipids				
2-Arachidonoyl-1-palmitoyl-sn-glycero-3-phosphocholine	PC(20:4/16:0)	781.5622	781.5615	-0.9
1,2-Dioleoyl-sn-glycero-3-phosphocholine	PC(18:1/18:1)	785.5935	785.5924	-1.4
1,2-Dioleoyl-sn-glycero-3-phosphocholine	PE(14:0/14:0)	635.4526	635.4541	2.4
1-Palmitoyl-sn-glycero-3-phosphocholine	PC(16:0/0:0)	495.3325	495.3338	2.6
Sterol Lipids				
Cholesterol	CHOL	386.3549	386.3556	1.8
Cholesteryl pelargonate	CE(9:0)	526.4750	526.4748	-0.4
Prenol lipids				
Coenzyme Q10	Coenzyme Q10	863.3435	863.3443	0.9
Squalene	SQE	410.7180	410.7185	1.2

Table S2. Summary of discriminated metabolites between the Control and HR group by LC-(+)ESIMS analysis.

m/z	RT (min)	*m/z*	RT (min)	*m/z*	RT (min)	*m/z*	RT (min)
202.1798	1.03	316.3203	1.86	548.4664	4.87	633.5052	17.88
102.1280	1.04	302.3047	1.86	564.4612	5.03	661.5364	19.63
118.0863	1.05	337.2341	1.87	558.4357	6.32	689.5677	20.73
316.2111	1.05	286.2734	1.90	568.4562	10.28	712.6435	21.35
114.0663	1.06	221.1168	2.00	526.5184	11.27	918.7527	21.62
218.2111	1.13	294.2058	2.00	758.5677	12.47	740.6746	21.80
100.0760	1.14	272.2942	2.13	781.5521	12.47	938.8148	21.94
246.2423	1.22	338.3020	2.20	780.5487	12.47	902.7265	21.94
502.2787	1.36	316.3202	2.22	848.5362	12.49	912.7993	21.95
446.2524	1.36	286.2734	2.31	784.5827	12.56	920.7677	21.95
432.2369	1.36	332.3151	2.62	806.5648	12.56	705.5809	22.04
362.3254	1.39	330.3358	2.73	554.5496	13.24	700.6256	22.04
318.2993	1.41	314.3046	2.81	742.5729	13.24	888.7985	22.14
274.2732	1.43	546.3990	3.10	734.5679	13.62	903.7387	22.18
296.2550	1.43	502.3728	3.19	782.5648	13.68	877.7231	22.19
230.2473	1.48	496.3388	3.25	786.5989	14.05	914.8147	22.19
231.2506	1.49	458.3467	3.27	808.5800	14.05	940.8303	22.21
415.2104	1.51	414.3205	3.35	876.5678	14.06	905.7540	22.42
119.0855	1.51	518.3204	3.55	680.4786	14.30	972.8571	22.42
281.1376	1.51	510.3545	4.01	707.4896	14.30	879.7385	22.42
460.2681	1.52	531.2731	4.12	663.4523	14.30	916.8305	22.42
474.2837	1.52	564.4611	4.12	778.5516	14.30	890.8079	22.43
135.0803	1.52	358.3671	4.18	327.0772	14.30	942.8458	22.44
437.1924	1.52	510.3543	4.35	686.4372	14.30	881.7542	22.63
453.1663	1.52	1041.6827	4.35	685.4338	14.30	918.8461	22.64
244.2629	1.57	532.3358	4.35	762.5990	15.26	907.7698	22.65
332.3149	1.58	600.3236	4.37	784.5800	15.26	944.8618	22.66
288.2889	1.60	540.4249	4.43	852.5678	15.27	671.5718	22.80
272.2578	1.64	679.4166	4.61	869.5582	15.28		
304.2839	1.81	695.3904	4.61	628.5499	17.88		

Table S3. Summary of discriminated metabolites between the Control and NDT2TD group by LC-(+)ESIMS analysis.

m/z	RT (min)	*m/z*	RT (min)	*m/z*	RT (min)	*m/z*	RT (min)
102.1280	1.04	314.3046	2.81	742.5729	13.24	903.7387	22.18
114.0663	1.06	546.3990	3.10	794.6035	13.26	877.7231	22.19
267.1220	1.36	502.3728	3.19	832.5801	13.75	898.7833	22.19
362.3254	1.39	522.3545	3.79	812.6125	14.01	929.7543	22.32
318.2993	1.41	532.3360	4.01	809.5837	14.05	900.7985	22.41
274.2732	1.43	510.3545	4.01	808.5800	14.05	875.7867	22.42
256.2629	1.43	531.2731	4.12	876.5678	14.06	905.7540	22.42
296.2550	1.43	564.4611	4.12	663.4523	14.30	879.7385	22.42
230.2473	1.48	358.3671	4.18	778.5516	14.30	916.8305	22.42
119.0855	1.51	510.3543	4.35	327.0772	14.30	862.7838	22.52
460.2681	1.52	1019.7008	4.35	762.5990	15.26	664.6013	22.53
437.1924	1.52	532.3358	4.35	785.5835	15.26	810.7525	22.53
453.1663	1.52	548.3098	4.36	784.5800	15.26	695.5720	22.59
244.2629	1.57	600.3236	4.37	800.5546	15.26	881.7542	22.63
332.3149	1.58	679.4166	4.61	852.5678	15.27	577.5178	22.63
288.2889	1.60	695.3904	4.61	869.5582	15.28	876.7993	22.63
272.2578	1.64	548.4664	4.87	788.6140	15.37	907.7698	22.65
304.2839	1.81	564.4612	5.03	811.6665	15.48	850.7839	22.65
316.3203	1.86	524.3701	5.21	813.6825	17.05	864.7993	22.76
302.3047	1.86	228.1954	5.94	617.5102	17.43	671.5718	22.80
286.2734	1.90	431.3835	5.95	685.4339	19.33	904.8303	22.88
221.1168	2.00	703.5733	12.06	647.4574	19.34	878.8150	22.90
322.2367	2.00	725.5546	12.06	703.5627	20.46	883.7703	22.90
294.2058	2.00	780.5487	12.47	687.5680	20.71	852.7994	22.92
272.2942	2.13	848.5362	12.49	894.7523	21.65	906.8463	23.17
316.3202	2.22	784.5827	12.56	902.7265	21.94		
286.2734	2.31	806.5648	12.56	920.7677	21.95		
332.3151	2.62	782.5651	12.60	705.5809	22.04		
330.3358	2.73	569.3800	12.77	927.7384	22.08		
520.3389	2.77	554.5496	13.24	888.7985	22.14		

Table S4. Summary of discriminated metabolites between the Control and HR group by LC-(–)ESIMS analysis.

m/z	RT (min)	*m/z*	RT (min)	*m/z*	RT (min)	*m/z*	RT (min)
225.0610	1.06	701.4284	4.58	856.6086	14.43	857.6768	17.00
367.1053	1.07	718.4187	4.58	856.6086	14.43	832.6647	17.19
242.0795	1.07	568.3625	5.18	857.6120	14.43	683.5955	19.78
393.2288	1.51	913.5855	8.01	750.5456	15.05	709.6114	19.92
395.2442	1.52	915.6009	8.15	1010.5548	15.22	692.6211	19.92
566.3469	3.77	1175.7760	11.95	806.5926	15.22	697.6112	20.36
556.3526	3.99	1176.7812	12.00	874.5800	15.22	680.6210	20.37
554.3463	4.32	955.5257	12.47	823.5828	15.22	694.6365	20.79
556.3523	4.32	1074.5105	12.50	891.5705	15.22	711.6268	20.79
571.3367	4.33	1023.5133	12.50	942.5674	15.22	684.6077	20.79
494.3257	4.33	746.5143	12.91	959.5574	15.22	762.6243	20.79
622.3342	4.33	878.5931	13.26	855.6609	15.44	686.6052	20.79
1063.6957	4.33	722.5144	13.39	855.6609	15.44		
639.3245	4.33	722.5144	13.39	923.6487	15.45		
707.3124	4.35	880.6082	13.74	925.6645	17.00		
653.4069	4.47	853.6449	13.99	993.6518	17.00		
655.4228	4.58	847.5825	14.02	857.6768	17.00		

Table S5. Summary of discriminated metabolites between the Control and NDT2TD group by LC-(–)ESIMS analysis.

m/z	RT (min)	*m/z*	RT (min)	*m/z*	RT (min)	*m/z*	RT (min)
247.0629	1.04	556.3523	4.32	722.5144	13.39	857.6768	17.00
255.8220	1.06	571.3367	4.33	880.6082	13.74	832.6647	17.19
113.0227	1.06	494.3257	4.33	853.6449	13.99	692.6211	19.92
160.8406	1.06	622.3342	4.33	847.5825	14.02	697.6112	20.36
179.0549	1.06	1063.6957	4.33	856.6086	14.43	680.6210	20.37
257.8191	1.06	639.3245	4.33	856.6086	14.43	694.6365	20.79
306.0879	1.06	707.3124	4.35	857.6120	14.43	711.6268	20.79
162.8377	1.06	653.4069	4.47	750.5456	15.05	762.6243	20.79
215.0321	1.06	655.4228	4.58	806.5926	15.22	686.6052	20.79
217.0291	1.06	701.4284	4.58	874.5800	15.22		
197.8070	1.06	568.3625	5.18	823.5828	15.22		
195.8099	1.06	913.5855	8.01	891.5705	15.22		
294.0526	1.06	1175.7760	11.95	942.5674	15.22		
293.0492	1.07	1176.7812	12.00	959.5574	15.22		
367.1053	1.07	955.5257	12.47	855.6609	15.44		
393.2288	1.51	1074.5105	12.50	855.6609	15.44		
395.2442	1.52	1023.5133	12.50	923.6487	15.45		
566.3469	3.77	746.5143	12.91	925.6645	17.00		
556.3526	3.99	878.5931	13.26	993.6518	17.00		
554.3463	4.32	722.5144	13.39	857.6768	17.00		

Table S6. The parameters of the LC-MRM-MS-based targeted metabolomic analysis in positive ion mode.

Q1 Mass (Da)	Q3 Mass (Da)	DP (V)	CE (eV)	Q1 Mass (Da)	Q3 Mass (Da)	DP (V)	CE (eV)
102	60	50	40	703.3	184	50	30
114	86	50	30	705.5	453.3	50	40
119	59	50	35	712.6	467.3	50	35
221	165	50	30	725.5	542.3	50	45
228.2	184	50	35	734.5	184.1	50	35
230.2	57	50	40	742.4	601.3	50	35
244.3	57	50	35	758.5	184.1	50	35
256.2	119	50	35	762.6	184.1	50	40
272.2	100	50	35	778.6	116	50	40
272.3	57	50	35	780.4	597.4	50	40
274.2	88	50	35	781.5	184.1	50	35
286.1	100	50	35	782.3	599.4	50	45
286.2	100	50	30	782.5	184	50	35
288.2	88	50	35	784.5	184.1	50	35
302.3	88	50	35	784.5	579.5	50	45
304.3	88	50	40	785.6	602.5	50	40
314.3	100	50	30	786.5	184.1	50	35
316.2	88	50	40	788.4	184.1	50	35
316.3	88	50	40	794.5	184.1	50	40
318.2	88	50	35	800.5	184.1	50	40
330.2	88	50	40	806.4	623.5	50	45
332.3	270.3	50	30	808.3	625.6	50	45
332.3	284.3	50	35	809.6	184	50	40
358.3	88	50	45	810.8	537.4	50	30
362.2	300.2	50	35	811.4	628.4	50	45
369	147	50	45	812.5	184	50	45
437.2	303	50	40	813.6	184	50	45
474.2	119	50	25	832.6	649.3	50	35
485	121	50	35	833.6	147	50	40
496.2	184.1	50	25	848.5	597.5	50	55
509.3	104	50	35	850.7	577.5	50	30
510.3	184	50	35	852.6	784.3	50	30
510.4	184	50	35	852.8	579	50	30
518.3	104	50	35	862.8	563.5	50	30
520.4	184	50	35	869.6	784.3	50	35
522.4	184	50	35	875.8	603.5	50	35
524.4	184	50	35	876.6	808.2	50	30
532.2	327.2	50	35	876.8	577.3	50	30
546.4	184.1	50	35	877.7	577.5	50	30

548.3	104	50	40	878.8	577.5	50	30
548.3	270.2	50	45	879.7	623.5	50	35
554.4	298.1	50	45	881.7	625.5	50	35
564.4	270.2	50	45	883.8	627	50	35
564.5	270.2	50	45	894.7	597	50	35
569.4	457.2	50	35	898.8	577.5	50	35
577.5	95	50	50	900.8	601.5	50	35
600.3	473.2	50	40	902.7	603.5	50	35
617	313	50	45	903.7	647.5	50	35
647.5	347.2	50	45	904.8	605.5	50	35
661.5	439.3	50	45	905.7	623.5	50	35
663.5	327	50	40	906.8	605.4	50	35
671.4	369.2	50	45	907.8	625.5	50	35
685.4	227	50	35	916.8	617.5	50	35
687.6	319.2	50	45	918.7	573.5	50	35
689.5	467.5	50	45	920.8	575.5	50	35
695.4	639.4	50	55	927.8	577.5	50	35
695.6	327.2	50	40	929.7	647.5	50	35
703.3	335	50	40				

Table S7. The parameters of the LC-MRM-MS-based targeted metabolomic analysis in negtive ion mode

Q1 Mass (Da)	Q3 Mass (Da)	DP (V)	CE (eV)	Q1 Mass (Da)	Q3 Mass (Da)	DP (V)	CE (eV)
225.1	131	-60	-35	709.6	663.6	-60	-35
367.2	97	-60	-45	634.6	378.4	-60	-45
393.2	257.1	-60	-45	697.6	62	-60	-40
395.2	135	-60	-45	648.5	392.5	-60	-45
566.3	281.3	-60	-35	711.4	62	-60	-40
494.3	269.3	-60	-35	571.3	62	-60	-40
554.3	269.3	-60	-35	853.6	793.6	-60	-35
556.2	270.2	-60	-40	847.6	62	-60	-40
556.3	270.2	-60	-40	857.6	797.5	-60	-35
622.3	113	-60	-30	832.7	772.6	-60	-35
653.4	277.2	-60	-40	823.6	746.5	-60	-35
568.3	283.2	-60	-45	564.3	279.2	-60	-35
913.4	605.4	-60	-65	769.4	241	-60	-40
915	241.1	-60	-60	745.5	685.4	-60	-35
746.4	327.2	-60	-40	747.5	687.5	-60	-35
878.6	327.2	-60	-40	764.4	687.6	-60	-40
722.4	303.2	-60	-40	885.4	303.2	-60	-60
880.6	329.3	-60	-40	854.6	794.5	-60	-35
856.6	305.3	-60	-40	838.5	778.4	-60	-35
750.5	303.3	-60	-40	922.5	113	-60	-35
806.6	269.4	-60	-40	801.6	741.4	-60	-35
874.6	113	-60	-40	858.6	305.2	-60	-50
855.6	795.5	-60	-35	924.4	113	-60	-35
923.6	795.5	-60	-40	832.4	772.6	-60	-35
925.5	797.5	-60	-40	656.6	113	-60	-35
857.6	797.5	-60	-35	815.5	113	-60	-40
620.5	364.4	-60	-45	857.5	797.5	-60	-35
646.6	392.4	-60	-45	871.5	811.6	-60	-35

The XCMS software package peak recognition, peak filtering, peak alignment and CAMERA analysis program based on R language applied in the research of serum lipomics of type 2 diabetes (taking LC - (+) ESI-MS spectral data as an example):

```
rm(list=ls(all=TRUE))
library(Biobase)
library(xcms)
library(multtest)
library(CAMERA)
sessionInfo()
xs<-xcmsSet(profmethod = "binlin",method="centWave",ppm = 2.5, peakwidth=c(5,30),
snthresh =20,prefilter=c(3,10000),integrate=1, mzdiff =0.02)
xs <-group(xs,bw=2,minfrac=0.8,mzwid=0.015)
save(xs,file="xs.Rda")
ret.xs.obiwarp <-retcor(xs,method="obiwarp",plottype="deviation")
ret.xs.obiwarp<-group(ret.xs.obiwarp, bw = 5,minfrac=0.8,mzwid=0.015)
ret.xs.obiwarp
fill.ret.xs.obiwarp<-fillPeaks(ret.xs.obiwarp)
fill.ret.xs.obiwarp
save(fill.ret.xs.obiwarp, file="fill.ret.xs.obiwarp.Rda")
an.1<-annotate(fill.ret.xs.obiwarp,sigma=6,perfwhm=0.3,cor_eic_th=0.75,maxcharge=3,
maxiso=3,mzabs=0.03,multiplier=3,polarity="positive")
peaklist.1<-getPeaklist(an.1)
write.csv(peaklist.1,file='annotated.1.csv')
an.2<-annotate(fill.ret.xs.obiwarp,sigma=6,perfwhm=0.3,cor_eic_th=0.75,maxcharge=3,
maxiso=3,mzabs=0.03,multiplier=3,polarity="positive")
peaklist.2<-getPeaklist(an.2)
write.csv(peaklist.2,file='annotated.2.csv')
an.3<-annotate(fill.ret.xs.obiwarp,sigma=6,perfwhm=0.3,cor_eic_th=0.75,maxcharge=3,
maxiso=3,mzabs=0.03,multiplier=3,polarity="positive")
peaklist.3<-getPeaklist(an.3)
write.csv(peaklist.3,file='annotated.3.csv')
an.4<-annotate(fill.ret.xs.obiwarp,sigma=6,perfwhm=0.3,cor_eic_th=0.75,maxcharge=3,
maxiso=3,mzabs=0.03,multiplier=3,polarity="positive")
peaklist.4<-getPeaklist(an.4)
write.csv(peaklist.4,file='annotated.4.csv')
report.fill.ret.xs.obiwarp<-diffreport(fill.ret.xs.obiwarp,"1","2",eicmax=8000,file="DB12
POS")
save(report.fill.ret.xs.obiwarp,file="report.fill.ret.xs.obiwarp.Rad")
report.fill.ret.xs.obiwarp<-diffreport(fill.ret.xs.obiwarp,"1","3",eicmax=8000,file="DB13
pos")
save(report.fill.ret.xs.obiwarp,file="report.fill.ret.xs.obiwarp.Rad")
report.fill.ret.xs.obiwarp<-diffreport(fill.ret.xs.obiwarp,"1","4",eicmax=8000,file="DB14
```

```
pos")
save(report.fill.ret.xs.obiwarp,file="report.fill.ret.xs.obiwarp.Rad")
report.fill.ret.xs.obiwarp<-diffreport(fill.ret.xs.obiwarp,"2","3",eicmax=8000,file="DB23
pos ")
save(report.fill.ret.xs.obiwarp,file="report.fill.ret.xs.obiwarp.Rad")
report.fill.ret.xs.obiwarp<-diffreport(fill.ret.xs.obiwarp,"2","4",eicmax=8000,file="DB24
pos")
save(report.fill.ret.xs.obiwarp,file="report.fill.ret.xs.obiwarp.Rad")
report.fill.ret.xs.obiwarp<-diffreport(fill.ret.xs.obiwarp,"3","4",eicmax=8000,file="DB34
pos")
save(report.fill.ret.xs.obiwarp,file="report.fill.ret.xs.obiwarp.Rad")
output <-groupval(ret.xs.obiwarp,value="into")
output2 <-t(output)
simca <-write.table(output2,sep=",","simca.csv")
```
